# The Heat Shock Transcription Factor HsfA Is Essential for Thermotolerance and Regulates Cell Wall Integrity in *Aspergillus fumigatus*

**DOI:** 10.3389/fmicb.2021.656548

**Published:** 2021-04-09

**Authors:** João Henrique Tadini Marilhano Fabri, Marina Campos Rocha, Caroline Mota Fernandes, Gabriela Felix Persinoti, Laure Nicolas Annick Ries, Anderson Ferreira da Cunha, Gustavo Henrique Goldman, Maurizio Del Poeta, Iran Malavazi

**Affiliations:** ^1^Departamento de Genética e Evolução, Centro de Ciências Biológicas e da Saúde, Universidade Federal de São Carlos, São Carlos, Brazil; ^2^Department of Microbiology and Immunology, Stony Brook University, Stony Brook, NY, United States; ^3^Laboratório Nacional de Biorrenováveis (LNBR), Centro Nacional de Pesquisa em Energia e Materiais (CNPEM), Campinas, São Paulo, Brazil; ^4^MRC Centre for Medical Mycology, University of Exeter, Exeter, United Kingdom; ^5^Faculdade de Ciências Farmacêuticas de Ribeirão Preto, Universidade de São Paulo, São Paulo, Brazil; ^6^Division of Infectious Diseases, School of Medicine, Stony Brook University, Stony Brook, NY, United States; ^7^Institute of Chemical Biology and Drug Discovery, Stony Brook University, Stony Brook, NY, United States; ^8^Veterans Administration Medical Center, Northport, NY, United States

**Keywords:** *Aspergillus fumigatus*, HsfA, thermotolerance, heat shock (HS), transcription factor, cell wall integrity (CWI)

## Abstract

The deleterious effects of human-induced climate change have long been predicted. However, the imminent emergence and spread of new diseases, including fungal infections through the rise of thermotolerant strains, is still neglected, despite being a potential consequence of global warming. Thermotolerance is a remarkable virulence attribute of the mold *Aspergillus fumigatus*. Under high-temperature stress, opportunistic fungal pathogens deploy an adaptive mechanism known as heat shock (HS) response controlled by heat shock transcription factors (HSFs). In eukaryotes, HSFs regulate the expression of several heat shock proteins (HSPs), such as the chaperone Hsp90, which is part of the cellular program for heat adaptation and a direct target of HSFs. We recently observed that the perturbation in cell wall integrity (CWI) causes concomitant susceptibility to elevated temperatures in *A. fumigatus*, although the mechanisms underpinning the HS response and CWI cross talking are not elucidated. Here, we aim at further deciphering the interplay between HS and CWI. Our results show that cell wall ultrastructure is severely modified when *A. fumigatus* is exposed to HS. We identify the transcription factor HsfA as essential for *A. fumigatus* viability, thermotolerance, and CWI. Indeed, HS and cell wall stress trigger the coordinated expression of both *hsfA* and *hsp90*. Furthermore, the CWI signaling pathway components PkcA and MpkA were shown to be important for HsfA and Hsp90 expression in the *A. fumigatus* biofilms. Lastly, RNA-sequencing confirmed that *hsfA* regulates the expression of genes related to the HS response, cell wall biosynthesis and remodeling, and lipid homeostasis. Our studies collectively demonstrate the connection between the HS and the CWI pathway, with HsfA playing a crucial role in this cross-pathway regulation, reinforcing the importance of the cell wall in *A. fumigatus* thermophily.

## Introduction

Fungal human diseases and their impacts on human health and world economy have frequently been overlooked (Brown et al., 2012). It is believed that fungal infections kill more than 1.5 million people worldwide per year (Benedict et al., 2019), and prospects are even worse if the last decades’ environmental changes are considered. For instance, it has been proposed that global warming may significantly enhance the adaptation of fungal populations to higher temperatures, which may cause the emergence of new fungal diseases associated with difficult treatment (Garcia-Solache and Casadevall, 2010; Casadevall et al., 2019).

In this worrisome scenario, the saprophytic mold and opportunistic human pathogen *Aspergillus fumigatus* stands out for its intrinsic thermophilic and thermotolerance traits and the rise of resistant isolates to available antifungal drugs (van Paassen et al., 2016). Unlike other *Aspergillus* species, *A. fumigatus* can germinate under temperatures above 40°C, and its conidia remain viable up to 70°C (Araujo and Rodrigues, 2004). These attributes of *A. fumigatus* biology help to explain its high prevalence in the environment and support the thermotolerance as an essential determinant for its pathogenicity since it allows the adaptation of the fungus to temperatures found before and after the infection of mammalian host and favors the persistence of this fungus inside the human lungs (Albrecht et al., 2010; Haas et al., 2016).

Living organisms continuously monitor the environmental temperature to survive (Leach and Cowen, 2013). In response to temperature rise, gene expression is adjusted, allowing the cells to synthesize a specific group of proteins called heat shock proteins (HSPs) that deal with this stressing condition (Parsell and Lindquist, 1993). During heat shock (HS), these molecular chaperones prevent the aggregation of denatured proteins and restore their native conformations and function (Hartl, 1996). To allow this orchestrated response, an influential group of conserved DNA binding proteins, the heat shock transcription factors (HSFs), regulate gene transcription during HS and other stress sources (Albrecht et al., 2010; Sueiro-Olivares et al., 2015; Gomez-Pastor et al., 2018). One member of this family, the heat shock transcription factor 1 (Hsf1), is a key player in mediating the cell transcriptional response to HS and has been widely studied in humans, *Saccharomyces cerevisiae*, and *Candida albicans* (Sarge et al., 1993; Eastmond and Nelson, 2006; Nicholls et al., 2009; Leach et al., 2016). Hsf1 is essential for viability and it is hyperphosphorylated in both fungal organisms in response to HS (Wiederrecht et al., 1988; Hashikawa and Sakurai, 2004; Nicholls et al., 2009, 2011). Under stressing conditions, Hsf1 homotrimers bind to specific DNA motifs named HS elements (HSE) located in target gene promoters, inducing the expression of molecular chaperones and other genes related to thermal adaptation (Sorger and Pelham, 1988; Sorger and Nelson, 1989; Yamamoto et al., 2005; Leach et al., 2016). Concomitantly, chaperones such as Hsp70 and Hsp90 are known regulators of Hsf1 activity via a well-described feedback regulatory loop in which Hsp90 binds to Hsf1 to keep it in an inactive state (Leach et al., 2012a; Schopf et al., 2017; Kijima et al., 2018; Krakowiak et al., 2018). In conditions where the chaperones are required for other functions and are no longer available for Hsf1 inhibition, the transcription of target HS genes increases (Veri et al., 2018). In *A. fumigatus*, putative HSEs were found in genes encoding HSPs and enzymes involved in the oxidative stress response, protein translation, carbohydrate and nitrogen metabolism, and signal transduction (Albrecht et al., 2010).

Thermoadaptation has also been directly associated with the activation of signaling pathways that govern cellular processes such as fungal morphogenesis and dimorphism, plasma membrane fluidity, and cell wall integrity (CWI) [reviewed in Brown et al. (2010); Leach and Cowen (2013); Fabri et al. (2020)]. The CWI pathway, which comprises the apical kinase PkcA, the three-component mitogen-activated protein kinases (MAPKs) Bck1, Mkk2, and MpkA, and the transcription factor RlmA (Valiante et al., 2009; Rocha et al., 2015, 2016), is one of the signaling cascades responsible for the regulation of cell wall biosynthesis and maintenance (Valiante et al., 2015). This signaling pathway also sustains the fungal cell wall viability during heat stress (Kamada et al., 1995; Fuchs and Mylonakis, 2009; Rocha et al., 2020b). However, despite the significance of the interplay between thermotolerance and CWI, little is known about the molecular events that reciprocally govern these two essential *A. fumigatus* virulence attributes.

Precedent exists indicating that the Hsf1-Hsp90 circuit is a connecting point between thermotolerance and the fungal CWI pathway (LaFayette et al., 2010; Lamoth et al., 2012; Leach et al., 2012a). For instance, without the proper expression of Hsp90 driven by Hsf1 in *S. cerevisiae* and *C. albicans*, CWI is compromised, leading to deficient expression of genes that promote CWI during exposure to high temperatures (Truman et al., 2007; Nicholls et al., 2009; Leach et al., 2012a). Also, Hsp90 potentiates the resistance to echinocandins in different fungal pathogens, including *A. fumigatus*, since depletion of this chaperone increases the susceptibility to these antifungals (Cowen, 2009; LaFayette et al., 2010; Lamoth et al., 2013, 2015; Chatterjee and Tatu, 2017). We have previously demonstrated that three critical components of the *A. fumigatus* CWI pathway (PkcA, MpkA, and RlmA) are Hsp90 clients (Rocha et al., 2020b), suggesting an involvement of the Hsf1-Hsp90 regulatory function over the *A. fumigatus* CWI pathway. However, it remained unclear how *hsfA^*HSF*1^* contributes to thermal adaptation and the disturbed cell wall homeostasis observed in the CWI pathway mutants. This is a follow-up study to our previous work (Rocha et al., 2020b), and our results show that cell wall ultrastructure is severely modified when cells are exposed to HS. We observe that *hsfA* is essential for thermotolerance, impacts the expression of genes related to the cell wall biosynthesis, and genetically interacts with the main players of the CWI and HOG pathway. We show a clear link between the HS and cell wall stress modulated by HsfA and the CWI pathway in *A. fumigatus* biofilms *in vitro* by following the fluctuations in HsfA expression. Our results also show that cell integrity genes are modulated when *hsfA* expression is repressed.

## Materials and Methods

### Strains and Culture Conditions

The *A. fumigatus* strains used in this study are described in Supplementary Table 1. Strains were maintained in complete medium [YG; glucose 2% (w/w), 0.5% yeast extract (w/w), 1 × trace elements)] or minimal medium [MM; glucose 1% (w/w), 1 × high-nitrate salts, and 1 × trace elements (pH 6.5)]. Trace elements and high nitrate salt compositions were as described previously (Malavazi and Goldman, 2012). For solid media, agar 2% (w/w) was added. To grow the ΔKU80 *pyrG1* strain, the media was supplemented with 1.2 g/L of uridine and uracil. When required, pyrithiamine (Sigma) or hygromycin B (Merck) was added to a final concentration of 0.2 μg/ml or 200 μg/ml, respectively. Different xylose concentrations were used depending on the experiment to induce *hsfA* expression in the *xylP::hsfA* conditional strain.

The wild-type and *xylP::hsfA* strains were grown and analyzed by DIC (Differential Interference Contrast) microscopy to analyze hyphal growth. Accordingly, 1 × 10^5^ conidia of each strain were inoculated in glass-bottom dishes (MatTek Corporation) containing 2 ml of MM supplemented with different xylose concentrations at 37°C for 12 h. Images were captured with an AxioCam MRm camera (Zeiss) and processed using ZEN software.

### Construction of the *A. fumigatus* Mutants

For the *xylP::hsfA* cassette construction, two fragments spanning the 5′ UTR region of *hsfA* (Afu5g01900) gene were PCR-amplified from genomic DNA of the CEA17 strain, according to Supplementary Figure 1A. The primers used are listed in Supplementary Table 2. The opposite sites of the 5′ regions contained a short sequence homologous to the multiple cloning site of the pRS426 plasmid (the small bold letters indicated in Supplementary Table 2). The *pyrG* gene inserted into the cassette was amplified from pCDA21 plasmid (Chaveroche et al., 2000) and used as a prototrophy marker. The pYES-hph-pXyl devR vector was used to amplify the *Penicillium chrysogenum* xylose reductase gene promoter (*xylP*), which is activated in the presence of xylose and repressed in the presence of glucose (Zadra et al., 2000). The substitution cassette was generated by *in vivo* recombination in *S. cerevisiae*, as reported previously (Malavazi and Goldman, 2012). All the PCR amplifications were performed using Phusion High-Fidelity DNA Polymerase (Thermo Scientific). The cassette was transformed into protoplasts of the *A. fumigatus* ΔKU80 pyrG1 according to previously described procedures (Malavazi and Goldman, 2012). Transformants were carefully tested by PCR (Supplementary Figure 1B) and Southern blot analysis, using the 5′ flanking region as a probe (Supplementary Figure 1C). An endogenous *hsfA* promoter region spanning 467 bp was maintained before the *hsfA* gene because we previously observed that complete replacement of the native *hsfA* promoter resulted in transformants with growth defects and inconsistent control of *hsfA* transcription (data not shown).

To generate the double mutant Δ*mpkA xylP::hsfA*, the *mpkA* deletion cassette was amplified from the genomic DNA of the Δ*mpkA* strain (Valiante et al., 2009) using primers MpkA 5F and MpkA 3′ REV and transformed into the *xylP::hsfA* strain selected for pyrithiamine resistance. The *hsfA* locus in this strain was checked using the primers HsfA 600 ups and HsfA 2 5UTR REV pRS426 (Supplementary Figure 1D), while the *mpkA* replacement was checked by using the primers MpkA FW and MpkA REV (Supplementary Table 2 and Supplementary Figure 1E). Likewise, to construct the double mutant *pkcA*^*G*579*R*^ *xylP::hsfA*, the *xylP::hsfA* cassette was transformed into the *pkcA*^*G*579*R*^ *pyrG-* strain (Rocha et al., 2020b). The *xylP::hsfA* replacement in this mutant was checked using the primers HsfA 5 FW and xylP REV (Supplementary Figure 1F), while the primers pkcA GC FW and Afu5g11970 3R (Supplementary Figure 1G) were used to check the mutated *pkcA* locus. The *pkcA*^*G*579*R*^ mutation is a Gly579Arg substitution in the PkcA protein, and the strain carrying this mutation is defective in the activation of MpkA and consequently the activation of the CWI pathway, resulting in altered expression of genes encoding cell wall-related proteins (Rocha et al., 2015). To construct the double mutant Δ*sakA xylP::hsfA*, the *sakA* deletion cassette was amplified from the genomic DNA of the Δ*sakA* strain (Altwasser et al., 2015) using primers sakA yes FW and sakA yes REV and transformed into the *xylP::hsfA* strain selected for hygromycin resistance. The *xylP::hsfA* locus in this mutant was checked using the primers HsfA 5 FW and xylP REV (Supplementary Figure 1H), while the primers IM-563 and IM-566 (Supplementary Figure 1I) were used to check the *sakA* locus.

To generate HsfA fusion with the luciferase gene (*luc*), a substitution cassette was constructed in which the *hsfA* genomic sequence without stop codon was cloned in-frame with the *luc* gene in a C-terminal fusion (Supplementary Figure 1J). The *luc* gene (1665 bp) without spacer was PCR-amplified from the pUC57 plasmid (Jacobsen et al., 2014) using the primers Luc FW and Luc REV. The *pyrG* gene was also used as a marker for prototrophy. The cassette was constructed *in vivo* in *S. cerevisiae* and transformed in *A. fumigatus* wild-type (Supplementary Figures 1K,L) and *pkcA*^*G*579*R*^ *pyrG-* (Supplementary Figures 1M,N) strains. The Δ*mpkA* cassette was also used to transform the *hsfA::luc* strain to obtain the double mutant (Supplementary Figures 1O,P).

To generate the reporter strain in which the *hsp90* promoter (*hsp90P*) was fused with the luciferase gene (*luc*), a substitution cassette was constructed by cloning the *hsp90P* (975 bp) in-frame with the *luc* gene (Supplementary Figure 1Q) flanked by 5′ UTR (0.833 kb) and 3′ UTR (0.573 kb) regions of the *pyrG* gene to allow integration of the construct at the *pyrG* locus. *hsp90P* was amplified from the genomic DNA of the CEA17 strain using the primers Hsp90P pyrG FW and Hsp90P RV luc, and the 5’ UTR of *pyrG* was amplified by the primers pRS426 5UTR pyrG FW and 5UTR pyrG RV. The fragment containing the *luc* gene, the hygromycin resistance marker gene, and the 3′ UTR region of *pyrG* was amplified from the pNB04 plasmid (Rocha et al., 2016) using primers luc 2 FW and pyrG 3UTR pRS426 RV. The *hsp90P::luc* cassette was generated by recombination in *S. cerevisiae* and transformed into the *A. fumigatus* wild-type (Supplementary Figures 1R,S), *pkcA*^*G*579*R*^ (Supplementary Figures 1T,U), Δ*rlmA* (Supplementary Figures 1T,V), and Δ*mpkA* (Supplementary Figures 1T,W) strains to obtain relevant single and double mutants.

### DNA Manipulation and Southern Blot Analysis

Southern blot analysis was used to confirm that the *xylP::hsfA* cassette integrated homologously at the targeted locus. Genomic DNA from *A. fumigatus* was extracted as previously described (Malavazi and Goldman, 2012). For Southern blot analysis, *Xho*I-restricted chromosomal DNA fragments were separated on a 1% agarose gel and blotted onto Hybond N^+^ nylon membranes (GE Healthcare), following standard techniques. Probe labeling was performed using AlkPhos Direct Labelling and Detection System (GE Healthcare) according to the manufacturer’s description. Labeled membranes were exposed to ChemiDoc XRS gel imaging system (BioRad) to generate the images.

### Transmission Electron Microscopy (TEM) Analysis

A total of 1 × 10^7^ conidia of the wild-type strain were statically grown in 10 ml of liquid YG for 36 h at 30°C. HS was induced by transferring the mycelia to fresh pre-heated YG (48°C) for 5, 10, 15, 30, and 60 min of incubation at 48°C. The control was left at 30°C. To analyze the cell wall organization in the conditional *hsfA* mutant, wild-type and *xylP::hsfA* strains were statically grown in liquid MM supplemented with xylose 1% for 36 h at 30°C. The mycelia were collected by centrifugation, washed twice with fresh MM, and further incubated in MM (repression) or MM lacking glucose but supplemented with xylose 0.06% (induction) for 4 h at 30°C. Finally, the repressed samples were heat-shocked at 48°C for 15, 30, and 60 min or treated with 2 μg/ml of caspofungin (CASP) for 1 h to induce cell wall stress. The untreated control remained at 30°C. Next, the mycelia were fixed in 3% EM grade glutaraldehyde in 0.1 M sodium cacodylate buffer, pH 7.4, for 24 h at 4°C. Cells were processed as described previously (Munshi et al., 2018), and TEM image acquisition was achieved using a FEI TeCnai12 BioTwinG2 microscope at an acceleration voltage of 120 kV. Digital images were acquired with an AMT XR-60 CCD Digital Camera system. Cell wall thicknesses of 50 sections of different germlings were measured using ImageJ software.

### Phenotypic Assays

The radial growth of the wild-type and *xylP::hsfA* strains at different temperatures was analyzed by spotting 1 × 10^4^ conidia of each strain onto the center of 90 mm Petri dishes containing solid MM supplemented with different concentrations of xylose. The plates were incubated for 72 h at 30°C, 37°C, or 48°C and analyzed. Similarly, to investigate the susceptibility of the strains to agents that impair cell wall maintenance or cell membrane [caffeine (CAF), calcofluor white (CFW), congo red (CR), caspofungin (CASP), and sodium dodecyl sulfate (SDS), respectively], 1 × 10^4^ conidia of each strain were inoculated on solid MM supplemented with xylose 0.06% and increasing concentrations of the drugs. The same procedures were employed to test susceptibility to osmotic stress caused by sorbitol and oxidative stress induced by paraquat and menadione. All the Petri dishes were incubated at 37°C for 72 h. As an alternative protocol, the susceptibility to H_2_O_2_, diamide, and the crop fungicide fludioxonil was investigated in 96-well plate assays containing 200 μl of liquid MM supplemented with xylose 0.06% and different concentrations of the agents mentioned above.

For the genetic analysis of double and single mutants, 10-fold dilution series (1 × 10^5^–1 × 10^2^) of the conidia were spotted on Petri dishes containing MM supplemented with different xylose concentrations and CASP. The plates were incubated at 30°C, 37°C, or 48°C for 48 h and analyzed.

### Luciferase Activity Assay

Luminescence quantification of the relevant strains was performed as described elsewhere (Rocha et al., 2016), with minor modifications. For the luciferase activity assay during HS, 2 × 10^5^ conidia of each strain were cultured in white, clear bottomed 96-well plates (Greiner Bio-one) containing 200 μl of MM supplemented with yeast extract 0.006% and grown at 30°C for 12 h for initial biofilm formation. Next, luciferin (0.5 mM) was added to each well and the plates were read initially at 30°C and incubated at 37°C or 48°C for 2 h. Luminescence readings were taken at 2-min intervals. For the cell wall stress assay, the same procedures were followed except that the plates were cultured at 37°C for 5 h. Subsequently, 2 μg/ml of CASP were added to each well along with luciferin 0.5 mM. The plates were incubated at 37°C for 4 h, and readings were recorded at 2-min intervals. All the luciferase activity experiments were read in Luminescence mode on the SpectraMax M5 (Molecular Devices). Normalization was made by the number of conidia. Mean ± SEM are shown in the graphs.

### RNA Extraction and RT-qPCR Procedures

To evaluate the *hsfA* expression in the conditional *xylP::hsfA* mutant, 1 × 10^8^ conidia of the wild-type and *xylP::hsfA* strains were grown for 24 h at 37°C in 50 ml of liquid MM supplemented with xylose 1% and transferred to fresh MM or MM without glucose supplemented with different concentrations of xylose (0.06, 0.2, 1, and 5%) for additional 4 h of growth. HS stress induction was achieved by incubating 1 × 10^8^ conidia of the wild-type strain in MM for 24 h at 30°C. Subsequently, mycelia were transferred to fresh pre-heated MM (48°C) for 2, 5, 10, 15, 30, 60, and 120 min of incubation at 48°C. The control was left at 30°C. Cell wall stress was achieved by incubating 2 × 10^7^ conidia of the wild-type strain in YG for 16 h at 37°C. Subsequently, the samples were subjected to CASP or CR exposure for 60 or 30 min, respectively. Mycelia from each culture condition were collected via vacuum filtration, frozen in liquid nitrogen, and stored at −80°C until used for RNA extractions.

The total RNA was extracted with Trizol reagent (Thermo Scientific) according to the manufacturer’s protocol. RNA was processed as described previously (Rocha et al., 2015). DNAse-treated total RNA from each strain was reverse-transcribed with High-Capacity cDNA Reverse Transcription kit (Thermo Scientific) as described elsewhere (Rocha et al., 2015). RT-qPCR was conducted with Power Sybr Green PCR Master Mix (Thermo Scientific). The primers for the individual genes were designed using Primer Express 3.0 software (Life Technologies) and are listed in Supplementary Table 3. RT-qPCR was performed in duplicate from three independent biological samples in a StepOne Plus Real-time PCR System (Thermo Scientific). The fold change in mRNA abundance was calculated using 2^–ΔΔ*Ct*^ equation, and all the values were normalized to the expression of β-tubulin (*tubA*).

### RNA-Sequencing

To induce heat stress, 1 × 10^8^ conidia from wild-type and *xylP::hsfA* strains were incubated in 50 ml of liquid MM supplemented with xylose 1% for 24 h at 30°C. Subsequently, mycelia were washed twice with MM and incubated for 4 h at 30°C in MM for *hsfA* repression (Baldin et al., 2015). HS was induced by transferring the mycelia to fresh pre-heated MM for 15 and 60 min at 48°C. The control was left at 30°C. Mycelium from each time point for both pre- and post-stress exposure was collected via vacuum filtration, immediately frozen in liquid nitrogen, and stored at −80°C until used for RNA extraction.

Total RNA was extracted using Trizol reagent, treated with DNase I (Qiagen) and purified using the RNeasy kit (Qiagen), according to the manufacturer’s instructions. RNA from each treatment was quantified using NanoDrop (Thermo Scientific) and analyzed on an Agilent 2100 Bioanalyzer system to assess RNA integrity (RIN = 9.0–9.5). Sample preparation and library construction were performed as described previously (Alves de Castro et al., 2016) using Illumina TruSeq stranded mRNA sample preparation kit. Libraries were quantified on Step One Plus equipment (Applied Biosystems) and sequenced (2 × 100 bp) on a HiSeq 2500 instrument. Short reads were submitted to the NCBI’s Short Read Archive under the Bioproject PRJNA690780. Obtained fastq files were quality checked with FastQC^1^ and cleaned with Trimmomatic (Bolger et al., 2014). Ribosomal RNA was removed using SortMeRNA (Kopylova et al., 2012), and high-quality reads were mapped to the *A. fumigatus* Af293 genome sequence using Tophat2 (Kim et al., 2013) in strand-specific mode. The saturation of sequencing effort was assessed by counting the number of detected exon–exon junctions at different subsampling levels of the total high-quality reads, using RSeQC (Wang et al., 2012). All samples achieved saturation of known exon–exon junctions. To assess transcript abundance, exonic reads were counted in a strand-specific way using the Rsubread library (Liao et al., 2013) from the Bioconductor suite (Huber et al., 2015). Calling of differentially expressed genes was carried out in EdgeR (Robinson et al., 2010), controlling for an FDR of 0.01. The fold changes and the statistical significances of all genes for each comparison are shown in Supplementary Table 4. Gene ontology (GO) enrichment analysis was performed using the KOBAS tool kobas.cbi.pku.edu.cn (Xie et al., 2011). Cluster analysis of the differently expressed genes was performed using hierarchical clustering in Multiple Experiment Viewer (MeV) software^2^.

## Results

### Heat Stress Affects the Structure of *A. fumigatus* Cell Wall

**FIGURE 1:**
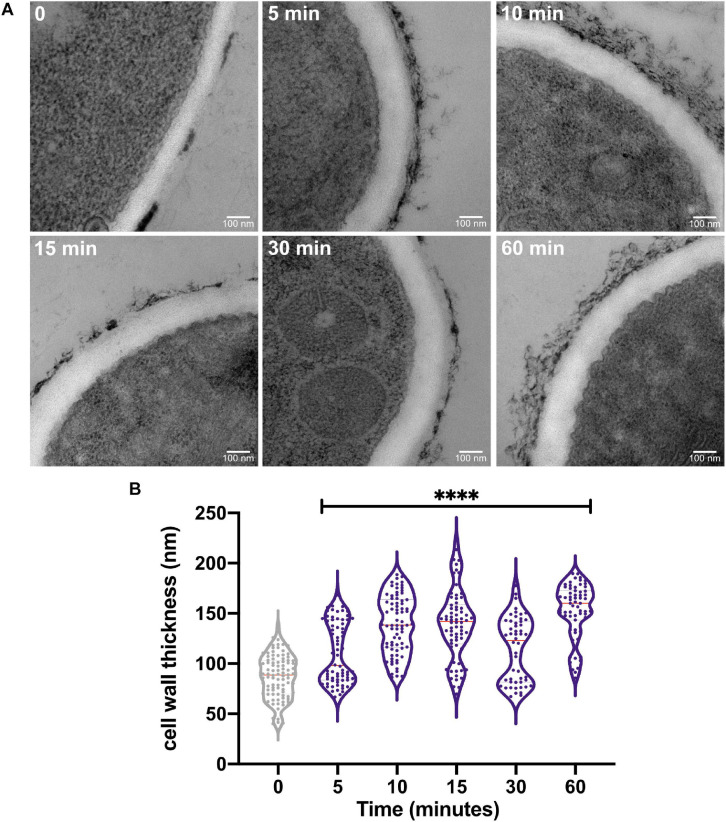
The heat shock stress causes an increase in the cell wall thickness of *A. fumigatus*. **(A)** The *A. fumigatus* wild-type strain was statically grown in complete liquid media for 36 h at 30°C, heat-shocked at 48°C for the indicated times, and prepared for transmission electron microscopy analysis. **(B)** Cell wall thickness measurement of the germlings shown in violin plot of 50 sections of different germlings. Dashed lines indicate the quartiles and red lines indicate the median for each point. ^****^p ≤ 0.0001 (one-way ANOVA and Dunnett’s multiple comparisons test).

Our previous results indicated that the *A. fumigatus* CWI pathway mutants are less tolerant to HS and PkcA signaling is required for early adaptation to HS (Rocha et al., 2020b). To gain insights about the early and prolonged effects of HS on the cell wall, we further analyzed the cell wall organization in the wild-type strain before and after HS at 48°C using TEM (Figure 1A). We observed a remarkable increase in the thickness of the fungus cell wall after HS exposure. Surprisingly, this increase is noticeable as early as after 5 min of exposure to high temperature resulting in a 27% increase in the cell wall thickness. Consistently, this increase is more evident after 10–15 min (60% increase) and 60 min (75% increase) of HS (Figure 1B). These results suggest that the sudden increase in temperature triggers the cell wall remodeling, indicating that this structure is highly dynamic and responsive to fluctuations in the surrounding temperature, potentially underlying a survival mechanism to counteract HS represented by the dramatic expansion of the cell wall.

### The *hsfA* Gene Is Essential for Thermotolerance and Required for Cell Wall Stress Resistance in *A. fumigatus*

The dramatic and quick changes in the cell wall architecture observed above when cells are challenged with HS prompted us to look for the impact of known regulators of thermoadaptation on the CWI of *A. fumigatus*. We have previously shown that the CWI pathway is activated when the cells are exposed to elevated temperatures and that the molecular chaperone Hsp90 interacts with the main components of this signaling pathway, assisting both the adaptation to HS and cell wall stress (Rocha et al., 2020b). Since, in eukaryotes, the *HSP90* gene expression is tightly governed by the transcription factor Hsf1 and this protein is one of the most important regulators of the HS response (Leach et al., 2016; Schopf et al., 2017), we searched for the *hsf1* homolog in *A. fumigatus*. A BLASTp search of the *A. fumigatus* A1163 genome database using *S. cerevisiae* and *C. albicans HSF1* as queries revealed a single open reading frame with significant similarity. The potential homolog, Afu5g01900 (hereafter named *hsfA*), shares 30% identity and 51% similarity (e-value 5e–26) with the ScHsf1 and 27% identity and 46% similarity (e-value 9e–29) with the CaHsf1. Furthermore, HsfA shows significant identity with Hsf1 of *A. nidulans* and *Aspergillus niger* (70% identity and 79% similarity, e-value 0.0; and 74% identity and 83% similarity, e-value 0.0, respectively). The HsfA region with the highest identity comprises the HSF-type DNA-binding domain of both yeast and human proteins (Veri et al., 2018), from residues 150 to 252 (2.8e-28; Pfam 00447).

**FIGURE 2:**
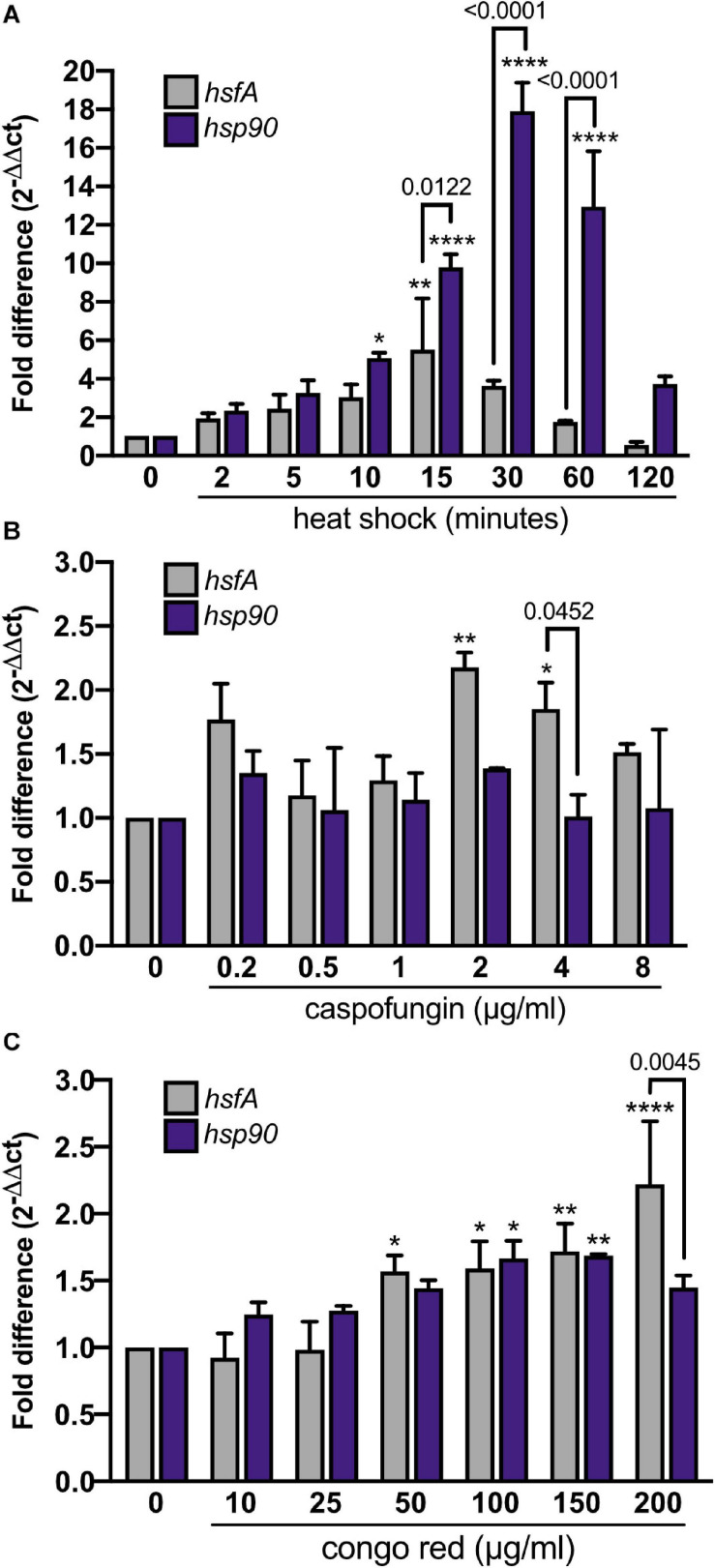
*hsfA* and *hsp90* expression respond to heat shock and cell wall stress. Expression of *hsfA* and *hsp90* was investigated by RT-qPCR. **(A)** The wild-type strain was grown in MM for 24 h at 30°C and heat-shocked for the indicated time points (min) at 48°C. The wild-type strain was grown in YG for 16 h at 37°C, and cell wall stress was achieved using increasing caspofungin concentrations for 60 min **(B)** or congo red for 30 min **(C)**. The fold difference of each gene represents the normalized total mRNA in relation to the same gene in the control condition. Mean ± SD (*n* = 3) are shown. Significant differences were observed by using two-way ANOVA followed by Sidak’s posttest. *p < 0.05, **p < 0.01, and ****p < 0.0001 indicate significant differences from comparisons to the same gene at the control condition. Differences between the genes are indicated by the bars.

As a first approach to study the role of HsfA in *A. fumigatus*, we analyzed the expression of *hsfA* in the *A. fumigatus* wild-type strain during HS and cell wall stress. We also assessed the mRNA levels of the *hsp90* gene, which is one of the most significant transcriptional targets of Hsf1 in eukaryotes (Erkine et al., 1999; Leach et al., 2016; Prodromou, 2016). We found that both *hsfA* and *hsp90* mRNA accumulation were rapidly induced in response to HS (Figure 2A), with *hsfA* reaching maximum expression after 15 min and *hsp90* after 30 min post-HS treatment compared to the control condition (30°C). After reaching the peak, *hsfA* mRNA levels decrease over time while high *hsp90* expression levels are sustained post-60 min of HS. These results are consistent with the role of *hsfA* as a transcription factor potentially upregulated when Hsp90-mediated *hsfA* inhibition is relieved upon HS, culminating with subsequent HsfA-mediated enhanced transcription of HSP, including Hsp90. When cells were challenged with cell wall stress induced by the addition of increasing concentrations of CASP, *hsfA* mRNA levels were significantly increased in the presence of 2 μg/ml and 4 μg/ml of the drug compared to the non-treated control condition (Figure 2B). Curiously, the *hsp90* accumulation under these conditions did not significantly change in comparison to the non-treated control. A similar expression profile for both genes was observed when different CR (congo red) concentrations were employed (Figure 2C), where an increase in *hsfA* mRNA abundance was detected at CR concentration ranging from 50 μg/ml to 200 μg/ml. These results indicate that *hsfA* is required to cope with the cell wall stress, but without a dramatic increase in mRNA *hsp90* abundance, suggesting a different regulatory mechanism for this TF in the presence of cell wall damage compared to temperature stress.

To further explore the role of *hsfA* in the *A. fumigatus* HS and cell wall stress response, we next attempted to generate an *hsfA* null mutant. Various deletion strategies and constructions were not successful, and after repeated attempts, no positive transformants were obtained (data not shown), suggesting that under standard laboratory conditions, *hsfA* is essential for viability in *A. fumigatus*, as observed for other fungal species, such as *S. cerevisiae* and *C. albicans* (Wiederrecht et al., 1988; Nicholls et al., 2009). Subsequently, we decided to generate a conditional *xylP::hsfA* mutant, in which the *hsfA* gene is under the control of the *P. chrysogenum xylP* promoter (Zadra et al., 2000). The substitution cassette contained the *pyrG* gene as the prototrophy marker, fused to the *xylP* promoter containing the first 467 bp of the native *hsfA* promoter (Supplementary Figure 1A). Diagnostic PCR and Southern blot analysis confirmed a single integration event in the conditional mutant (Supplementary Figures 1B,C).

**FIGURE 3:**
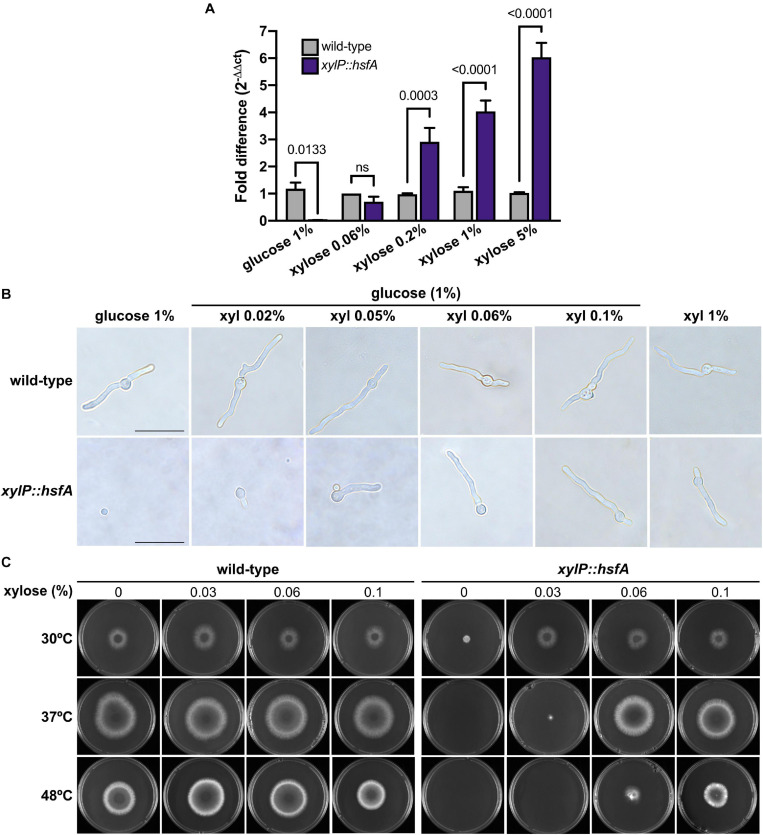
*hsfA* is essential for vegetative growth and thermotolerance. **(A)** *hsfA* expression in the wild-type and conditional lethal *xylP::hsfA* strain. The strains were grown for 24 h at 37°C in liquid MM supplemented with xylose 1% and transferred to fresh MM or MM lacking glucose supplemented with different xylose concentrations for 4 h. The fold difference of each condition represents the normalized mRNA abundance in comparison to the wild-type strain. Mean ± SD (*n* = 3) are shown. Statistics are indicated by the bars and *p* values are depicted (ns: non-significant; two-way ANOVA and Sidak’s multiple comparison test). **(B)** *hsfA* is required for *A. fumigatus* germination. The wild-type and *xylP::hsfA* strains were cultivated for 12 h at 37°C in glass-bottom dishes containing 2 ml of MM (glucose 1%) supplemented with increasing concentrations of xylose (xyl), or in xylose 1% as the sole carbon source. Magnification 100×; bar = 10 μm. **(C)** Radial growth of the wild-type and *xylP::hsfA* strains under repressive (glucose) or inducing conditions (xylose). A total of 1 × 10^4^ conidia of wild-type and *xylP::hsfA* strains were inoculated into the center of solid MM plates supplemented with the indicated concentrations of xylose and incubated at 30°C, 37°C, or 48°C for 72 h.

To validate the conditional expression of *hsfA* in this mutant, the mRNA abundance of *hsfA* was determined by RT-qPCR, both in the presence of glucose 1% (MM) for repression and in MM supplemented with increasing concentrations of xylose for *xylP* promoter induction (Figure 3A). The *hsfA* expression levels in the conditional lethal mutant increased in a xylose concentration-dependent manner (up to 5%), while in the wild-type strain, it remained constant. The results showed that at low xylose concentration (0.06%) *hsfA* mRNA abundance in the *xylP::hsfA* mutant was similar to that recorded for the wild-type strain. Consistently, *hsfA* expression was significantly suppressed in the absence of xylose.

In order to evaluate if *hsfA* repression causes any changes in the germination and structure of *A. fumigatus* hyphae such as lysis, which could be consistent with cell wall defects, an optical microscopy experiment was conducted in which both the wild-type and the *xylP::hsfA* strains were cultivated in MM containing different concentrations of xylose for 12 h at 37°C. As expected, the absence of xylose completely inhibited growth of the conditional mutant without noticeably affecting germination of the conidia (Figure 3B). In contrast, the *xylP::hsfA* mutant started germination with an increase in xylose concentration, achieving a comparable wild-type growth with 0.06 to 0.1% of xylose in the medium. These results indicate that HsfA is essential for *A. fumigatus* viability. Except for germination inhibition at low xylose concentrations, no change in the mutant hyphae morphology was observed. For comparison, in a medium lacking glucose and supplemented with xylose 1% as the sole carbon source, the conditional mutant grew normally, without the emergence of any hyphae structural alteration (Figure 3B).

Because thermotolerance is a key feature for the *A. fumigatus* biology (Bhabhra and Askew, 2005) and HsfA may be a major regulator of this attribute, we initially attempted to understand the role played by *hsfA* in ensuring thermotolerance. The wild-type and the *xylP::hsfA* strains were cultured in MM supplemented with varying xylose concentrations at different temperatures (Figure 3C). Although under basal condition (30°C), the *xylP::hsfA* strain under repressive condition showed decreased radial growth compared to the wild-type strain, growth was comparable to that of the wild-type strain when a small concentration of xylose was added to the medium (0.03%). However, at 37°C, the conditional mutant could not grow under repressive conditions, further suggesting the crucial role of *hsfA* for thermoadaptation of the fungus. The radial growth of the mutant was equivalent to the wild-type strain in the presence of at least 0.06% xylose. For this reason, this concentration of xylose was chosen for the next phenotypic assays below. The *xylP::hsfA* mutant susceptibility was even higher at 48°C since it could only grow properly when more than 0.1% of xylose was added to the medium (Figure 3C). Altogether, these results suggest that the cell viability is highly dependent on *hsfA* upon temperature increase.

**FIGURE 4:**
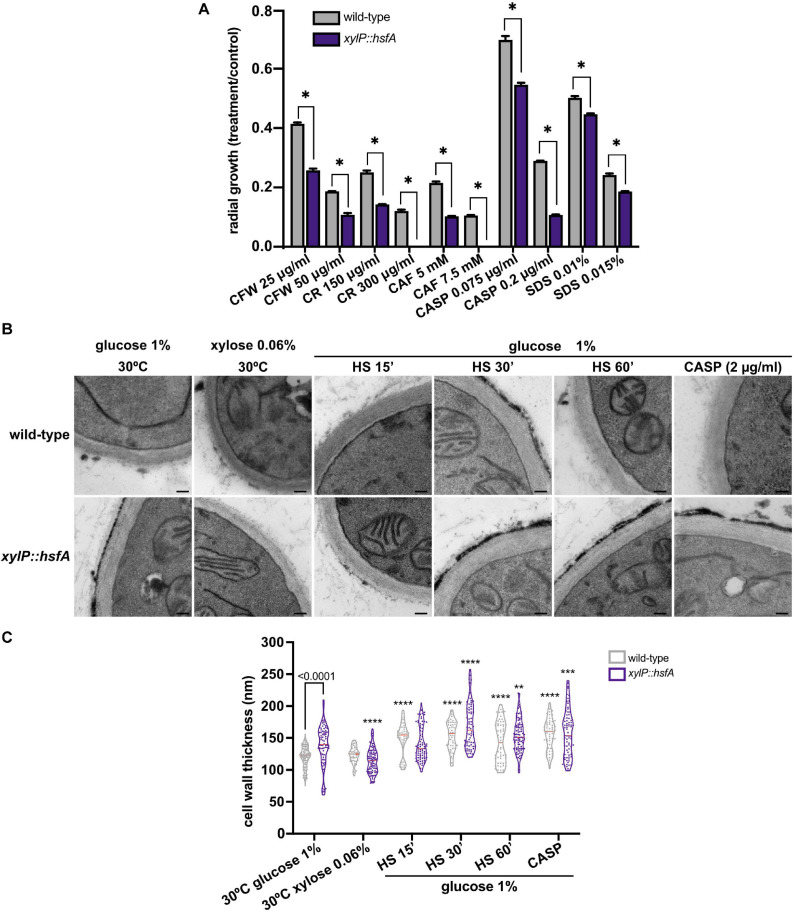
The *xylP::hsfA* mutant is more susceptible to cell wall stress. **(A)** A total of 1 × 10^4^ conidia of the wild-type and *xylP::hsfA* strains were inoculated onto solid MM supplemented with 0.06% xylose (low *hsfA* expression conditions) and varying concentrations of calcofluor white (CFW), congo red (CR), caffeine (CAF), caspofungin (CASP), and sodium dodecil sulfate (SDS). The plates were incubated at 37°C for 72 h and the ratio of radial growth of treated to the control condition was calculated. The results were expressed as mean ± SD, *n* = 3. *p ≤ 0.0001 (two-way ANOVA and Sidak’s posttest). **(B)** HsfA depletion increases cell wall thickness in *A. fumigatus*. The wild-type and *xylP::hsfA* strains were grown in liquid MM supplemented with 1% xylose for 36 h at 30°C and further incubated in MM (repression) or MM lacking glucose supplemented with xylose 0.06% (induction) for 4 h at 30°C. Next, the repressed samples were heat-shocked at 48°C for 15, 30, and 60 min or treated with 2 μg/ml of CASP for 60 min, and prepared for transmission electron microscopy analysis. Black bars: 100 nm. **(C)** Cell wall thickness measurement of the germlings shown in violin plot of 50 sections of different germlings. Dashed lines indicate the quartiles and red lines indicate the median for each condition. Significant differences were observed by using two-way ANOVA followed by Sidak’s posttest. **p < 0.01, ***p < 0.001, and ****p < 0.0001 indicate significant differences from comparisons to the same strain at the control (30°C, glucose 1%) condition. Differences between the strains in the same growth condition are indicated by the bars.

As our qPCR analysis hinted at the importance of *hsfA* as an adjunctive transcription factor required to cope with cell wall stress, we next investigated the relevance of *hsfA* for cell wall maintenance by evaluating the susceptibility of the conditional lethal mutant when grown in low *hsfA* expression level conditions (0.06% xylose, Figure 3). We observed differences in the conditional mutant susceptibility profile compared to the wild-type strain in the presence of cell wall stressing agents. Among the drugs tested, the highest growth inhibition was recorded for CASP, CAF, and CR treatments (Figure 4A). For these experiments, small increments in the amount of xylose added to the culture media caused recovery of the lethal phenotype, thus making it difficult to determine a xylose concentration to balance viability of the mutant and repression of *hsfA* to assess susceptibility to these cell wall stressors. Thus, we suggest that the narrow range of xylose concentration that results in a dramatic difference in growth (for instance, compare xylose concentration of 0.03 and 0.06% in Figure 3C) can partially explain the subtle cell wall phenotypes we present here.

Nevertheless, the results shown in Figure 4A point to a role of *hsfA* in cell wall homeostasis. In line with this evidence, when the *xylP::hsfA* conditional lethal mutant was grown under repressive conditions (MM) and analyzed by TEM, we observed a significant cell wall thickening (15%) at non-HS basal condition (30°C), which was fully recovered when 0.06% xylose was added to the culture medium (Figures 4B,C), further suggesting that *hsfA* plays a crucial role controlling the expression of cell wall-related genes. As expected, the cell wall thickness of the wild-type strain increased during the HS and during cell wall stress caused by CASP. In contrast, the cell wall thickening of the *xylP::hsfA* conditional mutant was similar to the wild-type strain under the two stress conditions, although significantly different from the control condition (1% glucose, 30°C), where the cell wall is constitutively thicker. These results suggest that *hsfA* is required for typical cell wall architecture under basal conditions, while *hsfA* loss of function retains the ability to remodel the cell wall ultrastructure when challenged with CASP and HS.

In addition to temperature stress tolerance, Hsf1 in yeast is also induced by oxidative stress, ethanol exposure, and glucose starvation (Liu and Thiele, 1996; Hahn and Thiele, 2004; Hashikawa et al., 2007). Consistently, the *xylP::hsfA* strain showed increased sensitivity to all oxidative stress compounds tested (menadione, hydrogen peroxide, and diamide) in comparison to the wild-type strain, except for paraquat (Supplementary Figure 2). The conditional mutant was also more susceptible to the crop fungicide fludioxonil (Supplementary Figure 2B). In contrast, there were no growth differences between the conditional mutant and the wild-type strain in the presence of osmotic stress caused by high concentrations of sorbitol (data not shown). These results suggest a role for *hsfA* in oxidative stress detoxification in *A. fumigatus*.

### *HsfA* Genetically Interacts With the MAPKs *mpkA* and *sakA*

Previous reports suggested that *A. fumigatus* cell wall undergoes continuous remodeling in response to environmental stimuli or stress conditions (Rocha et al., 2020a). Under these circumstances, the biosynthesis and reinforcement of the cell wall rely on the concerted actions of the CWI pathway and the High Osmolarity Glycerol (HOG) pathway since both cascades have overlapping functions to promote adaptation to cell wall-targeting and temperature stresses (Altwasser et al., 2015; Rocha et al., 2015, 2020b; Bruder Nascimento et al., 2016). Also, the MAPK cascades associated with these circuits crosstalk with the Hsf1 transcription factor through the action of the Hsp90 in *S. cerevisiae* and *C. albicans* (Lamoth et al., 2012; Leach et al., 2012a). As *hsfA* repression caused cell wall phenotypes, we asked whether this transcription factor genetically interacts with the apical kinase (*pkcA*) and the MAPK (*mpkA*) of the CWI pathway and the MAPK of the HOG pathway (*sakA*). Accordingly, double conditional mutants of *xylP::hsfA* with *pkcA*^*G*579*R*^ allele and Δ*mpkA*, or Δ*sakA* were constructed (Supplementary Figures 1D–I).

**FIGURE 5:**
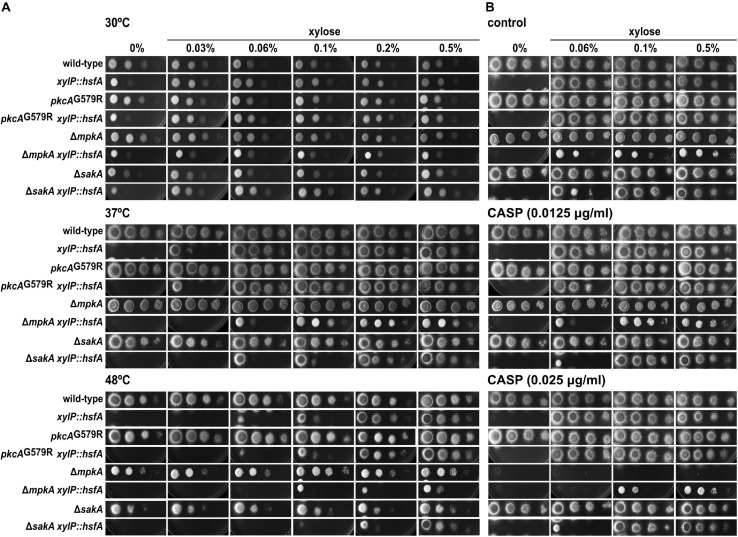
*hsfA* genetically interacts with *mpkA* and *sakA* during temperature and cell wall stresses. Tenfold dilution series of conidia from the wild-type, *xylP::hsfA*, *pkcA*^*G*579*R*^, *pkcA*^*G*579*R*^ *xylP::hsfA*, Δ*mpkA*, Δ*mpkA xylP::hsfA*, Δ*sakA*, and Δ*sakA xylP::hsfA* strains was inoculated into solid MM containing different concentrations of xylose. **(A)** Plates were incubated at 30°C, 37°C, or 48°C for 48 h. **(B)** 0.0125 μg/ml and 0.025 μg/ml of caspofungin (CASP) were used to induce cell wall stress and the plates were incubated at 37°C for 48 h.

Phenotypic tests with these double mutants were conducted under low xylose concentration to titrate out the expression of *hsfA* and score genetic interactions when *hsfA* is limiting inside the cell, since the recovery of *hsfA* expression occurs in largely low xylose concentration, as mentioned above (Figure 3). We observed that *mpkA* and *sakA* genetically interact with *hsfA* upon growth at 37°C and 48°C since the respective double conditional mutants grew less at lower xylose concentrations than the parental strains (Figure 5A). The same results were observed during cell wall stress caused by CASP (Figure 5B), CR, and CFW (Supplementary Figures 3A,B), as well as in the presence of SDS (Supplementary Figure 3C). Consistently, when the xylose concentration is increased to 0.1–0.5%, growth of the double conditional mutant is completely rescued, indicating that restoration of *hsfA* expression to wild-type levels is sufficient to fully recover the phenotype in the absence of either *mpkA* or *sakA*. These results also point out that *mpkA* and *sakA* are not essential for *hsfA* activation during thermoadaptation and cell wall stress.

### HsfA and Hsp90 Act Together to Endure Cell Wall Stress and HS via the CWI Pathway

Given the above reported synthetically sick genetic interactions between *hsfA* and the MAPKs of the CWI and HOG pathways, we used the luciferase (*luc*) reporter gene to investigate differences in HsfA protein expression during HS and cell wall stress induced by CASP. The rationale behind this experiment was to examine a role of these MAPKs in regulating posttranscriptional levels of HsfA. Hence, single and double mutants containing HsfA protein controlled by its native promoter and C-terminally fused to the luciferase protein were constructed (Supplementary Figures 1J–P). All the reporter strains, with the exception of Δ*sakA hsfA::luc*, showed no growth defects compared to the parental strains, indicating that the replaced alleles are fully functional (Supplementary Figure 4). Since the viability and growth of several Δ*sakA hsfA::luc* transformation mutants were impaired under HS conditions compared to the parental strains (data not shown), the evaluation of the *hsfA* expression was not possible in this mutant.

**FIGURE 6:**
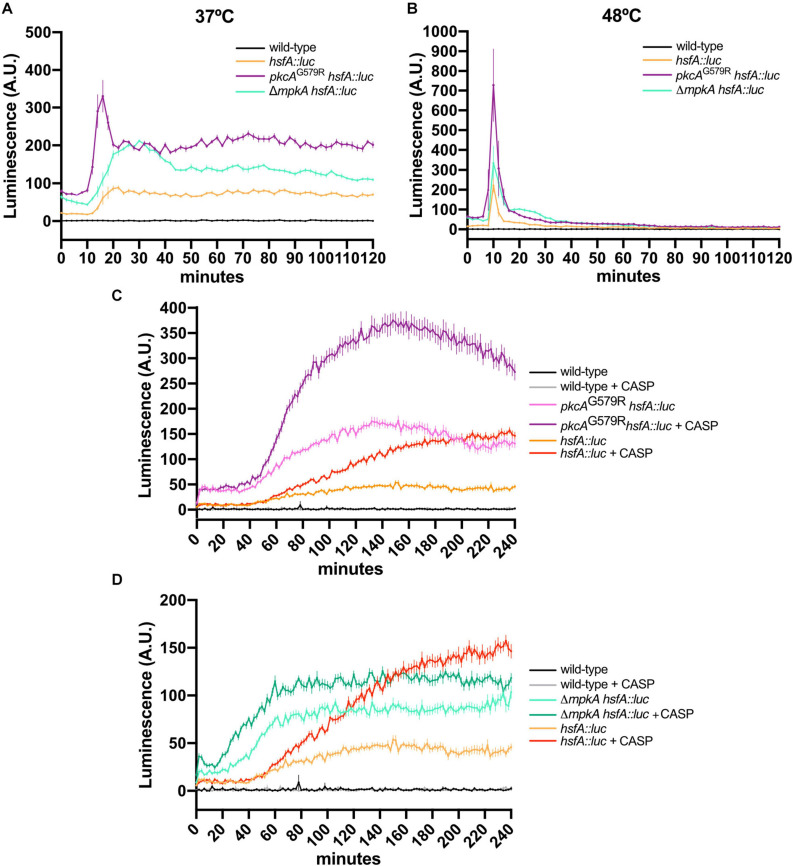
The loss of function of CWI regulators increases HsfA expression in *A. fumigatus* biofilms under HS and cell wall stress. **(A,B)** Luciferase activity assay of the *hsfA::luc*, *pkcA*^*G*579*R*^ *hsfA::luc*, and Δ*mpkA hsfA::luc* strains during the HS at 37°C **(A)** and 48°C **(B)**. **(C,D)** Luciferase activity assay of the *pkcA*^*G*579*R*^ *hsfA::luc* **(D)** and Δ*mpkA hsfA::luc* strains during cell wall stress caused by CASP. The wild-type strain without luciferase gene was used as the negative control in all experiments. Mean ± SEM (*n* ≥ 8) are shown. The results were normalized by the number of conidia (2 × 10^5^ per assay) and are expressed as luminescence (arbitrary units). CASP: caspofungin (2 μg/ml).

The luciferase activity was recorded after exposing the germlings of the reporter strains to HS and CASP. We observed that HsfA protein expression was rapidly induced after 15 min of HS at both 37°C (Figure 6A) and 48°C (Figure 6B) in the biofilm of all strains. These results are consistent with the peak in mRNA abundance recorded under the same conditions for the wild-type strain (Figure 2A), thus validating our HsfA reporter strain. However, the luciferase signal was more intense in the strains carrying the *pkcA^*G*579*R*^* and Δ*mpkA* mutations at both temperatures. In these genetic backgrounds, sustained high HsfA levels were recorded at 37°C up to 120 min of HS. Although, at 48°C, the same HsfA expression peak was detected after 15 min, the luciferase signal returned to wild-type levels after the 30 min of HS adaptation. These results highlight the importance of *hsfA* and the CWI pathway in the thermoadaptation of *A. fumigatus* to the mammalian host environment.

The expression of HsfA was also significantly induced by CASP treatment after 60 min of drug exposure (Figures 6C,D), similarly to the mRNA *hsfA* levels recorded under the same conditions in the wild-type strain (Figure 2B). Interestingly, the HsfA high levels were maintained up to 240 min of treatment. Again, we observed a positive synergistic effect in the HsfA expression when the *pkcA*^*G*579*R*^ mutant allele was combined with the CASP exposure (Figure 6C). The same synergistic effect was also observed in the combination of CASP and the Δ*mpkA* mutant (Figure 6D). We also conclude that *pkcA* and *mpkA* are not required for HsfA expression, and the loss of function of these kinases enhances the accumulation of HsfA possibly via undescribed compensatory mechanisms to retain the CWI and thermotolerance.

**FIGURE 7:**
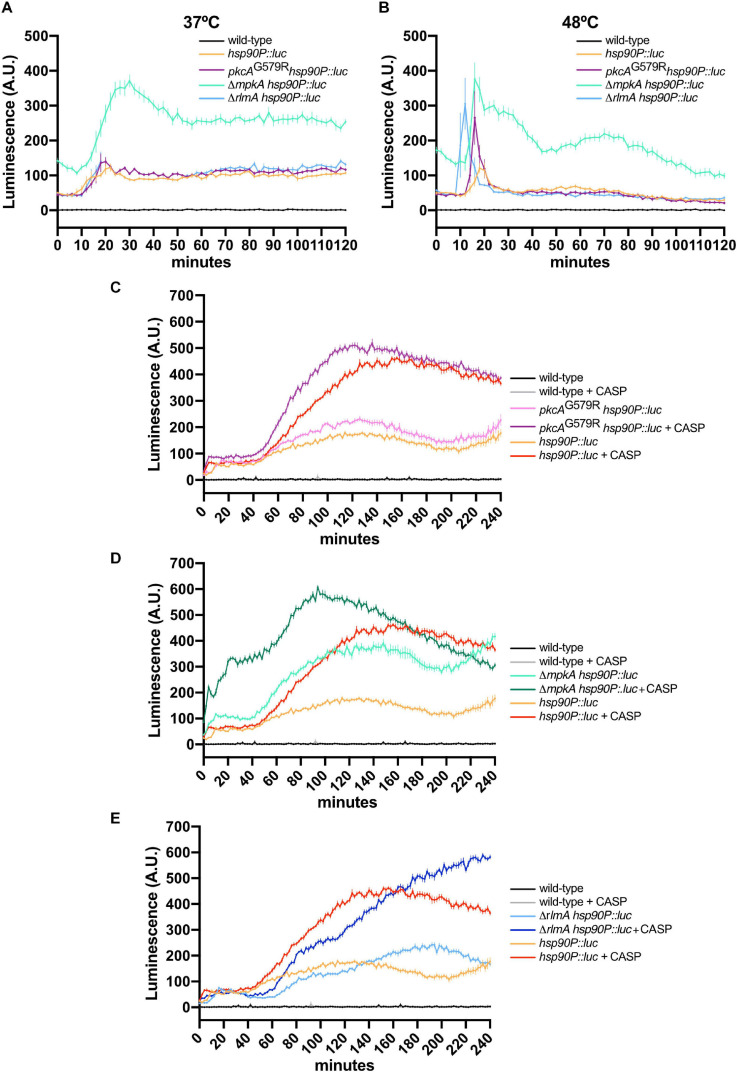
The loss of function of CWI regulators increases *hsp90* expression in *A. fumigatus* biofilms under HS and cell wall stress. **(A,B)** Luciferase activity assay of the *hsp90P::luc*, *pkcA*^*G*579*R*^ *hsp90P::luc*, Δ*mpkA hsp90P::luc*, and Δ*rlmA hsp90P::luc* strains during the HS at 37°C **(A)** and 48°C **(B)**. **(C–E)** Luciferase activity assay of the *pkcA*^*G*579*R*^ *hsp90P::luc* **(C)**, Δ*mpkA hsp90P::luc* **(D)**, and Δ*rlmA hsp90P::luc* **(E)** strains during cell wall stress caused by CASP. The wild-type strain without luciferase gene was used as the negative control in all experiments. Mean ± SEM (*n* ≥ 8) are shown. The results were normalized by the number of conidia (2 × 10^5^ per assay) and are expressed as luminescence (arbitrary units). CASP: caspofungin (2 μg/ml).

Given the *hsp90* levels are critical for the HsfA transcription and to probe further into the potential mechanisms underlying the enhanced HsfA expression in the CWI pathway mutants, we assessed the activity of the *hsp90* promoter (*hsp90P*) under the same conditions we used above to trace the HsfA protein accumulation. We constructed CWI pathway mutants in which the *hsp90P* was fused to the luciferase gene to generate relevant reporter strains (Supplementary Figures 1Q–W). For these experiments, we also included the deletion strain of the CWI pathway-associated transcription factor RlmA. As expected, *hsp90P* activity was rapidly upregulated during HS in the *hsp90P::luc* strain at 37°C (Figure 7A) and 48°C (Figure 7B), peaking at 15–20 min post-HS. At 37°C, there were no significant differences in the luminescence signal between the wild-type, *pkcA^*G*579*R*^*, and Δ*rlmA* backgrounds.

In contrast, the *mpkA* deletion strongly induced the *hsp90P* throughout the experiment, even under basal conditions (time point 0). At 48°C, a peak of *hsp90P* activity occurred earlier in all mutant strains compared to the wild-type reporter, and this effect was more evident in the Δ*rlmA* deletion background. Similar to the results scored at 37°C, the higher induction of *hsp90P* occurred in the *mpkA* deletion strain. Altogether, these data (Figures 6, 7) confirm our previous results that the HS response in the *A. fumigatus* biofilm is dysregulated when the CWI pathway is impaired (Rocha et al., 2020b) and reveal that *pkcA* and *mpkA* are also the primary sensors for the compensatory activation of both *hsfA* and *hsp90* expression.

Finally, *hsp90P* activity was also highly induced during the cell wall stress caused by CASP (Figure 7). Interestingly, this effect was similar for the *pkcA*^*G*579*R*^ and Δ*mpkA* mutants, which peaked after 100–120 min post-CASP treatment. However, the luminescence signal was higher in the Δ*mpkA* strain under basal conditions. In contrast, the luciferase signal increased later in the Δ*rlmA* strain and was kept at high levels after the signal of the *hsp90P* activity decreased in the other CWI pathway mutants (Figures 7C,D). Collectively, the luciferase assays indicate that the expression of HsfA and Hsp90 is connected, and both jointly work in response to HS and cell wall stress. Additionally, the results suggest a direct HsfA role in governing the expression of genes involved in the cell adaptation to these stressing conditions as noted by the compensatory increase in the HsfA expression in the CWI pathway mutants.

### HsfA Repression Modulates the Expression of Genes Related to Cell Wall Homeostasis and HS Response

In order to assess HsfA-dependent gene expression targets during HS, the wild-type and *xylP::hsfA* transcriptomes were investigated during HS exposure for 15 and 60 min at 48°C, after *xylP::hsfA* was repressed in MM lacking xylose, as described in the *Materials and Methods* section. The first time point (15 min) corresponds to the peak in *hsfA* expression at mRNA and protein levels (Figures 2A, 6B). The exposure of *A. fumigatus* for 60 min causes an overall 20% reduction of the metabolic activity of mature wild-type *A. fumigatus* biofilm (Rocha et al., 2020b) and does not cause loss of viability of the *xylP::hsfA* conditional lethal mutant when shifted to repressive conditions. Thus, these two time points allow us to identify early and late genes induced by HS.

**FIGURE 8:**
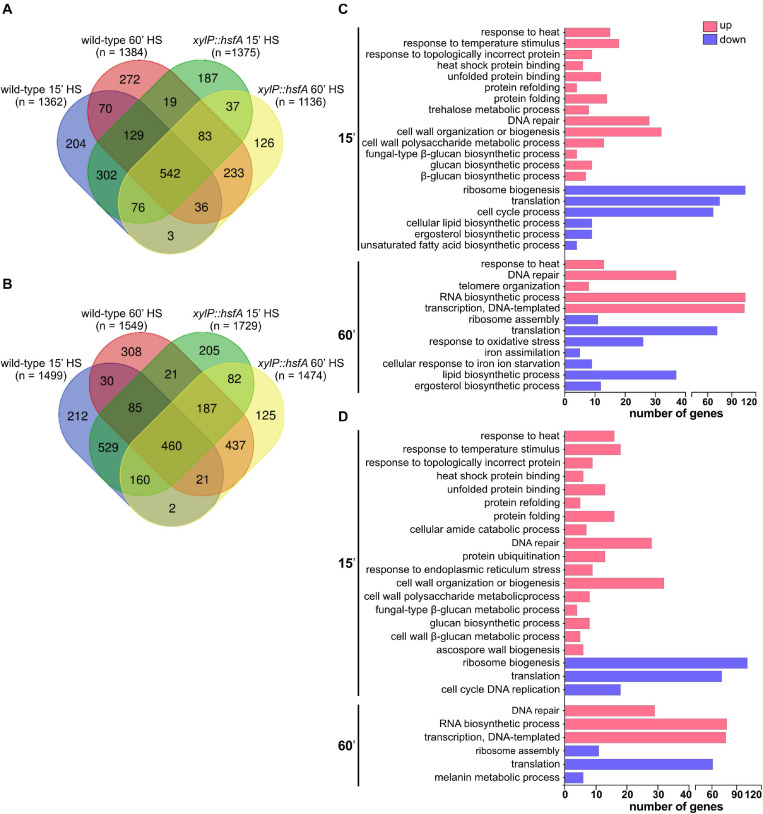
Global transcriptional response of the wild-type and *xylP::hsfA* strains to HS. Venn diagram depicting the number of upregulated (log_2_FC ≥ 1.0) **(A)** and downregulated (log_2_FC ≤ –1.0) **(B)** genes in the wild type and *xylP::hsfA* strains post-15 min and 60 min of HS. **(C)** Selected Biological Process Gene Ontology terms enriched from significantly differently expressed genes (log_2_FC ≥ 1.0 or log_2_FC ≤ –1.0) in the wild-type strain during HS (15 min and 60 min time points), in comparison to control condition (30°C). **(D)** Selected Biological Process Gene Ontology terms enriched from significantly differently expressed genes (log_2_FC ≥ 1.0 or log_2_FC ≤ –1.0) in the *xylP::hsfA* strain during HS compared to control condition (30°C). For the full Biological Process Gene Ontology terms list, refer to Supplementary Table 5.

HS elicited rapid modulation of gene expression since 1,362 genes were upregulated (log_2_FC ≥ 1.0) while 1,499 genes were downregulated (log_2_FC ≤ −1.0) in the wild-type strain post-15 min. After 60 min of HS, these numbers increased to 1,384 and 1,549, respectively (Figures 8A,B). In the *xylP::hsfA* strain under repression, 1,375 genes were upregulated (log_2_FC ≥ 1.0) while 1,729 genes were downregulated (log_2_FC ≤ −1.0) post-15 min of HS. After 60 min HS, these numbers decreased to 1,136 and 1,474, respectively (Figures 8A,B).

Intriguingly, the *xylP::hsfA* mutant showed a GO enrichment similar to the wild-type strain after 15 min of HS. Both strains demonstrated a transcriptional upregulation of genes involved in the cellular response to heat, protein folding, and refolding (Figures 8C,D), which are expected biological processes activated in response to temperature increase (Albrecht et al., 2010). Some of these genes were also differentially expressed at the protein level under HS, such as the chaperones Hsp90 (Afu5g04170), Hsp30 (Afu3g14540), Hsp70 (Afu1g07440), Hsp60 (Afu2g09290), Hsp78 (Afu1g11180), Hsp88 (Afu1g12610), Ssc70 (Afu2g09960), and Sti1 (Afu7g01860), as previously reported (Albrecht et al., 2010). The early HS response also caused significant upregulation of genes involved in glucan biosynthetic process and cell wall organization or biogenesis in both strains (Figures 8C,D), including the CWI pathway transcription factor *rlmA* (Afu3g08520); the catalytic subunit of the β-1,3-glucan synthase *fksA* (Afu6g12400); the β-1,3-glucanosyltransferases *gelA* (Afu2g01170) and *gel7* (Afu6g12410); the chitin synthases *chsE* (Afu2g13440), *chsF* (Afu8g05630), and *csmB* (Afu2g13430); and the MAPK kinase of the HOG signaling pathway *pbs2* (Afu1g15950). These results support the cell wall remodeling observed during HS (Figure 1). In contrast, only the wild-type strain showed enrichment of genes involved in trehalose biosynthesis (Figure 8C), suggesting the participation of HsfA in this process. At the same time, protein ubiquitination and the response to endoplasmic reticulum stress were processes enriched only in the repressed *xylP::hsfA* mutant (Figure 8D). Among the downregulated genes post-15 min of HS, there was an enrichment of ribosome biogenesis and translation processes in both the wild-type and mutant strains (Figures 8C,D), suggesting an abrogation of protein synthesis to sustain thermoadaptation. The GO analysis of downregulated genes also showed enrichment in the category involved in the ergosterol biosynthetic process and unsaturated fatty acid biosynthetic process in the wild-type strain (Figure 8C). These findings reflect the cellular balance to overcome increased membrane fluidity caused by temperature increase and suggest that HsfA has a role in lipid homeostasis and the balance in the content of saturated/unsaturated fatty acids ratio elicited by the HS.

After 60 min of HS, the upregulated genes in both strains were enriched for different metabolic processes, such as DNA repair, RNA biosynthetic process, and transcription, while the downregulated genes were involved in ribosome assembly and translation (Figures 8C,D). The profile of genes modulated after prolonged HS exposure excluded the categories involved in cell wall metabolism. Interestingly, only the wild-type strain retained an enrichment of upregulated genes involved in response to HS after 60 min, supporting the evidence of a late *hsfA* role during the HS. Again, the lipid and ergosterol biosynthetic processes were also found downregulated only in the wild-type strain. The iron assimilation and cellular response to iron ion starvation categories were also downregulated exclusively in the wild type (Figure 8C), which is in agreement with previous studies that have shown a relationship between Hsf1 and the cellular iron pool in *C. albicans* (Nair et al., 2017, 2018).

**FIGURE 9:**
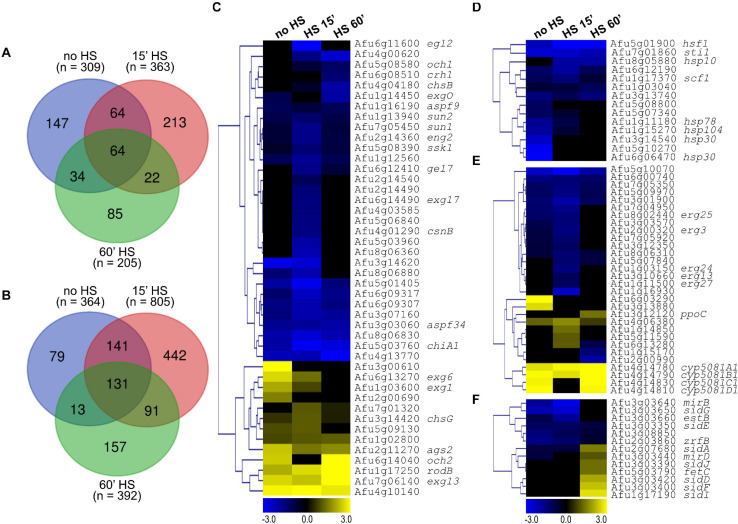
*hsfA* repression impacts the expression of genes related to HS, cell wall, lipid, and iron metabolism. Venn diagrams comparing the genes upregulated (log_2_FC ≥ 1.0) **(A)** and downregulated (log_2_FC ≤ –1.0) **(B)** in the *xylP::hsfA* mutant strain relative to the wild-type strain at no HS condition and post-15 min or 60 min of HS. **(C–F)** Hierarchical clustering analysis showing selections of differently expressed genes (log_2_FC ≥ 1.0 or log_2_FC ≤ –1.0). The heat maps are divided by processes and categories of interest: cell wall biosynthesis and organization **(C)**; heat shock response, chaperone activity, and protein folding **(D)**; lipid metabolism **(E)**; and iron metabolism **(F)**. All the heat maps were created in the Multiple Experiment Viewer (MeV) platform, using hierarchical clustering and Euclidean Distance with average linkage clustering.

Subsequently, to identify genes whose expression was directly influenced by *hsfA*, we compared the *xylP::hsfA* mutant grown on repressive conditions with the wild-type strain, and we observe that several genes are modulated either at the non-HS control condition (30°C) or during temperature stress (Figures 9A,B). Some of them were constitutively repressed or induced in the mutant strain, including the *hsfA* gene, which is repressed throughout the experiment, thus validating our experimental conditions (Supplementary Figure 5). Notably, four genes of the pyripyropene biosynthetic gene cluster (*pyr4*, *pyr5*, *pyr6*, and *pyr9*) were highly induced in the mutant strain. Consistently, other genes belonging to this secondary metabolite cluster, such as *pyr1* (Afu6g13920), *pyr3* (Afu6g13940), and *pyr8* (Afu6g14000), were upregulated both in the non-HS and at the 60-min time point (Supplementary Table 4). These data suggest that HsfA has a role in the biosynthesis of this meroterpenoid.

Focusing on the genes with participation on the cell wall organization, we observed few genes involved in the biosynthesis of cell wall, such as the α-1,3-glucan synthase *ags2* (Afu2g11270) and the chitin synthase *chsG* (Afu3g14420), which were upregulated, and β-1,3-glucanosyltransferase *gel7* (Afu6g12410) and the chitin synthase *chsB* (Afu4g04180), which were downregulated in at least one time point. In contrast, a number of genes involved in the remodeling of the cell wall were identified including the β-1,3-exoglucanases *exg1* (Afu1g03600) and *exg6* (Afu6g13270), the glycosyl hydrolase Afu4g13770, the chitinases *chiA1* (Afu5g03760), Afu5g03960 and Afu5g06840, and the glucanases *eg12* (Afu6g11600), *aspf9* (Afu1g16190), *exgO* (Afu1g14450), Afu1g12560, Afu8g06830, Afu2g14490, and Afu8g06360 (Figure 9C). Noteworthy, the more evident alterations in the mRNA abundance of most of these cell wall-related genes were a noticeable repression post-15 min of HS, when the expression of HsfA reaches its peak (Figures 2A, 6B), suggesting that *hsfA* has a role in the transcriptional activation of such genes and is important for cell wall metabolism during thermoadaptation.

As expected, many HSPs, such as *sti1* (Afu7g01860), *hsp10* (Afu8g05880), *scf1* (Afu1g17370), *hsp78* (Afu1g11180), *hsp104* (Afu1g15270), *hsp30/42* (Afu3g14540), *hsp20/26* (Afu5g10270) and *hsp30* (Afu6g06470), were repressed in the mutant strain in comparison to the wild type (Figure 9D), also indicating that they are putative HsfA transcriptional targets. In addition, several genes related to lipid metabolism, mainly those involved in ergosterol and fatty acids biosynthesis, were downregulated, especially at no HS condition and post-15 min of HS (Figure 9E), pointing to a rearrangement of the cellular plasma membrane to increase its fluidity in response to thermal insult (Leach and Cowen, 2014). Finally, siderophore metabolic genes were also considerably modulated and divided into two groups: one containing downregulated genes at basal condition and post-15 min of HS, and another comprising upregulated genes post-60 min of HS (Figure 9F). This last group coincides with the repression of the GO terms related to iron metabolism in the wild-type strain at that time point (Figure 8C). Independent RT-qPCR experiments validated the RNA-seq results for the six selected genes that represent the GO categories shown in Figure 9. We investigated the mRNA levels of *hsfA*, *hsp30*, *ags2*, *chsG*, *ppoC*, and *sidA* and presented the results as the ratio *xylP::hsfA*/wild-type (Supplementary Figure 6).

Collectively, our RNA-seq results strongly suggest that temperature stress causes major changes in the *A. fumigatus* transcriptome, and the transcription factor HsfA is a key regulator in the modulation of a significant number of these genes, being especially important for the response to HS, cell wall remodeling, plasma membrane homeostasis, and iron metabolism.

Given that thermotolerance and CWI are reciprocally controlled, and multiple transcription factors are intertwined to regulate these two polygenic biological processes, we analyzed our dataset to identify transcription factors differentially expressed in the wild-type and the *xylP::hsfA* strains exposed to HS. We concentrated our analysis in the transcription factors that presented differential expression values (log_2_FC ≥ 1.0 or log_2_FC ≤ −1.0) in at least one time point by comparing in this analysis the wild-type versus the *xylP::hsfA*. This search identified many transcription factors, including several of them uncharacterized (Supplementary Figure 7).

The putative transcription factors encoded by the genes Afu17060, Afu1g17150, Afu1g15370, Afu4g06420, and Afu4g07090 (*znfA*) were upregulated only in the mutant strain in at least one time point of HS, suggesting that they may play a role similar to that of HsfA during the HS response when *hsfA* is repressed. Concomitantly, Afu2g03020, Afu5g01662, Afu6g01840, Afu7g01820, and Afu1g01340 were genes upregulated only in the wild-type strain (Supplementary Figure 7), indicating that these genes may be direct or indirect regulated by HsfA. Afu7g06590, Afu3g06940, Afu8g05010 (*zpfA*), Afu1g11290, Afu5g00435, Afu5g10040, and Afu1g03210 (*flbD*) were among the downregulated genes only in the *xylP::hsfA* mutant strain in at least one time point of HS (Supplementary Figure 7). RgdA (Afu3g13920) is a transcriptional factor important for conidiation and cell wall architecture (Jun et al., 2020). Interestingly, in our analysis, this gene was considerably upregulated after 60 min of HS only in the wild-type strain, suggesting a possible target of HsfA regulatory network. Moreover, the gene encoding the transcription factor RttA (Afu7g04740) was also recently identified as playing a role in adaptation to the azole tebuconazole (Toyotome et al., 2020), and here it was downregulated in the wild-type strain post-60 min of HS. Altogether, the transcriptome analysis of transcription factors revealed probable additional regulators of the *A. fumigatus* HS response.

## Discussion

Although endothermy is a mammalian protective mechanism against fungal infections (Robert and Casadevall, 2009; Bergman and Casadevall, 2010), thermophilic fungi such as *A. fumigatus* can overcome this obstacle, indicating that thermotolerance in this fungus may be related to the expression of stress response genes that support its persistence inside the host. However, the cellular consequences of *A. fumigatus* adaptation to heat, the impact on the cell wall organization, and the participation of the signaling pathways that coordinate these two events are not fully understood.

**FIGURE 10:**
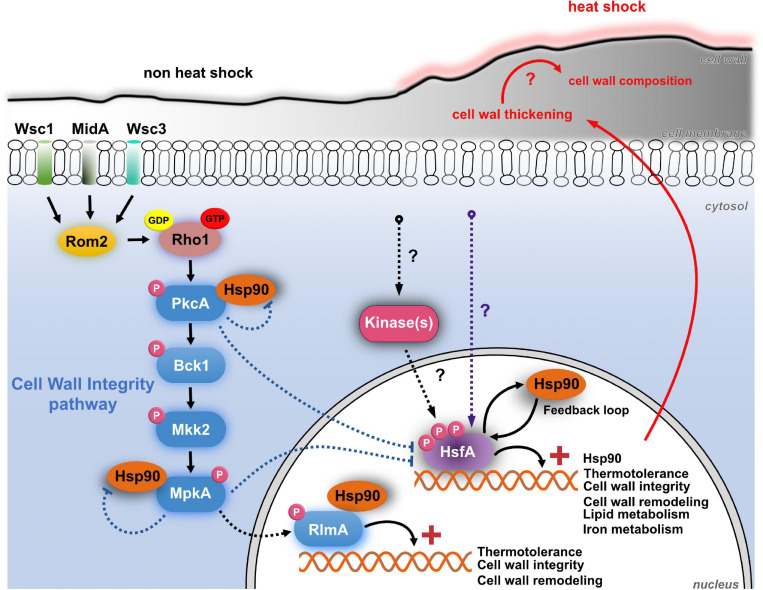
The crosstalk between HsfA-Hsp90 and CWI pathway in *A. fumigatus* during HS. The CWI pathway is activated during early adaptation to HS, possibly via MidA mechanosensor located at the cell membrane. This signal funnels into Rom2, a guanine nucleotide exchange factor, and the small Rho GTPAse Rho1, leading to the activation of PkcA and the CWI pathway. These events culminate with the phosphorylation of the MAP kinase MpkA, which, in turn, phosphorylates the transcription factor RlmA, thus activating the transcription of genes (**+**) important for thermotolerance, cell wall integrity, and remodeling. The CWI pathway proteins PkcA, MpkA, and RlmA are constitutive Hsp90 clients. A direct consequence of the HS is the thickening of the cell wall after 5 min of temperature increase, which may have consequences to the remodeling of the cell wall and exposure of carbohydrate to the cell surface. However, these consequences remain to be investigated (red question marks). Also, the cellular plasma membrane becomes more fluid in response to thermal insult. Although not necessary for HsfA activation, PkcA and MpkA negatively regulate the expression of HsfA and Hsp90, both during the HS (dashed blue arrows) and upon cell wall stress induced by caspofungin (not shown in the Figure; see text for details). The resulting increase in protein abundance of HsfA and Hsp90 in the *pkcA* and *mpkA* mutants possibly occurs via undescribed compensatory mechanisms accompanied by the activation of additional signaling cascades to retain the CWI and thermotolerance (purple dashed line). In the face of heat stress, mRNA abundance of HsfA increases and triggers the expression of genes related to thermotolerance, cell wall integrity, cell wall remodeling, lipid, and iron metabolism. One of its transcriptional targets, Hsp90, is also induced to deal with HS and assist the stabilization of client proteins. After about 15–30 min of HS, the HsfA levels decrease, possibly due to the action of Hsp90 in the regulatory feedback loop. This regulatory mechanism comprises the Hsf1/Hsp90 protein–protein interaction (not investigated here), which is reversed during the HS, when Hsp90 protein levels increase and decouple from Hsf1 to assist the folding of client proteins. The activity of Hsf1 remains until Hsp90 again binds to Hsf1, negatively regulating Hsf1 (see text for details). Hyperphosphorylation (not investigated here) is another regulatory event that modulates the transcriptional activity of HsfA orthologs in other fungal pathogens. The protein(s) kinase(s) responsible for HsfA phosphorylation are still unknown in *A. fumigatus*. This diagram is based on data from this article and the references (Dichtl et al., 2012; Leach et al., 2012b; Rocha et al., 2020a, b).

Recently, we demonstrated that in addition to the regulation of cell wall biosynthesis and maintenance, the CWI pathway activity is modulated during early regulation of *A. fumigatus* thermotolerance and showed that Hsp90 is necessary for this crosstalk by interacting with the mains players of the CWI signaling cascade (Rocha et al., 2020b). Hsp90 is fundamental for the eukaryotic HS response by promoting the folding and assembly of newly synthesized proteins (Taipale et al., 2010). Since the conserved transcription factor Hsf1 controls *HSP90* transcription (Sarge et al., 1993; Eastmond and Nelson, 2006; Nicholls et al., 2009; Leach et al., 2016), here we identified and characterized the putative *A. fumigatus HSF1* homolog and assigned its function in thermotolerance and the associate cell wall stress response, which we hypothesize is a key fungal response mechanism to heat (Figure 10). We revealed that HsfA is an essential transcription factor necessary for thermotolerance and vegetative growth, as observed in other fungi (Wiederrecht et al., 1988; Thompson et al., 2008; Nicholls et al., 2009; Yang et al., 2017).

Here, we provide strong evidence that an outcome of the heat stress is the exacerbated thickening of the cell wall in *A. fumigatus* (Figures 1, 4) accompanied by the induction of genes involved in cell wall biogenesis and remodeling (Figure 8). These results suggest that the cell wall expansion is necessary to sustain thermoadaptation without loss of viability. Increased cell wall thickness has also been recently reported in *C. albicans* biofilm hyphae during mild heat stress (Ikezaki et al., 2019). However, our results reveal that such modifications in cell wall architecture are very rapidly sensed by the cells since we detect a thickening of this structure as early as after 5 min of HS exposure (Figure 1). However, we should also consider that this rearrangement process in the cell wall observed at the initial times, such as after 5 min of HS, can be an event not exclusively directed by the activity of the CWI pathway or the transcriptional activity of HsfA. For instance, it could also be related to physical changes in the cell surface, such as increased Brownian movement of water molecules or expansion of proteins and polysaccharides in the cell wall. Additional experimentation is required to address this hypothesis. Nevertheless, these data point out that the morphological alterations of the cell wall during HS potentially result in modifications in the synthesis or exposure of specific sugars on the cell surface, e.g., α-glucan, β-glucan, chitin, mannoproteins, or galactosaminogalactan (Figure 10). Since the invading conidia germinate and eventually proliferate at high-temperature conditions inside the host, compared to the environmental niche, such cell wall adaptation may also exist during the initial steps of fungal infection and can be a determinant for immune recognition. However, the consequences of these alterations for the immune recognition of the fungus still need rigorous investigation. In *C. albicans*, repression of *HSP90* expression in a *tetO-HSP90* conditional mutant leads to increased detection of chitin by CFW fluorescence binding assay (Leach et al., 2012a; Nair et al., 2017). Likewise, depletion of Hsp90 in *A. fumigatus* caused increased sensitivity to cell wall stress (Lamoth et al., 2012).

Interestingly, our data demonstrate that the ability of the cell wall to be remodeled during the heat stress or CASP treatment was maintained in the *xylP::hsfA* mutant, indicating that this process is not entirely dependent on this transcription factor, even though the conditional lethal mutant *xylP::hsfA* is sensitive to cell wall-damaging agents, including CASP. However, under the non-HS condition (30°C), the repression of *hsfA* instantly causes a thickening of the cell wall (Figure 4), similar to the *C. albicans HSF1* (Nair et al., 2017) and *HSP90* conditional mutants (Leach et al., 2012a). In our case, we show that the cell wall thickening in the *xylP::hsfA* mutant is completely rescued if *hsfA* expression is restored by low xylose concentration, again reinforcing the crosstalk between resistance to heat and the cell wall structure. Despite the subtle increase in *hsfA* expression recorded in a wild-type strain challenged with CASP and CR (Figure 2), a peak in *hsfA* mRNA abundance occurred after 15 min of HS, coinciding with the higher thickening of the cell wall and the HS response establish by the concomitant increase in *hsp90* expression (Figures 2, 7). Similar patterns of gene expression are observed for other transcription factors, which are regulated posttranscriptionally and undergo minimal transcriptional changes. In this case, the intracellular pools of these transcription factors are likely present at all times in order to respond to stress quickly. A similar scenario may be the case for HsfA in the presence of cell wall stress. Whether posttranscriptional regulation of HsfA instead of transcriptional activation is a key event to deal with cell wall stress would require additional experiments, including investigation of the phosphorylation status and the activity of HsfA in response to cell wall-damaging agents. In line with this idea, very slight changes in xylose concentration can rescue the conditional lethal phenotype of *xylP::hsfA* mutant, suggesting that low levels of *hsfA* provide a survival benefit (Figures 3, 5).

Concomitantly to the dramatic changes in the cell wall structure, we observed increased expression of *hsp90* in response to *hsfA* upregulation, followed by a subsequent drop in *hsfA* mRNA accumulation (Figures 2, 6, 7), suggesting that the expected feedback loop between HsfA and Hsp90 described in *C. albicans* also occurs in *A. fumigatus* (Nicholls et al., 2009; Leach et al., 2012a, 2016). Consistently, the protein levels of Hsp90 remain high up to 240 min post-HS (Rocha et al., 2020b). In addition, HsfA was also shown to be important in controlling the expression of genes encoding HSPs (Figure 9) and enzymes involved in trehalose biosynthesis (Figure 8 and Supplementary Table 4), which together make up part of the canonical HS response (Takahashi et al., 2017). However, the genes encoding chaperones such as *hsp60*, *hsp70*, and *hsp90*, although modulated by HS, were not significantly impacted by the repression of *hsfA* (Supplementary Table 4), differently from what was observed in *C. albicans* (Nicholls et al., 2009; Leach et al., 2016). Possibly, the small increase in *hsfA* mRNA levels recorded in our RNA-seq samples under repressive conditions (1.5-fold; 15 min HS, Supplementary Table 4) could have elicited upregulation of these HSPs in the *xylP::hsfA* conditional mutant. The hypothesis that other transcription factors may be acting should also be considered, and Supplementary Figure 7 shows some possible candidates, such as *znfA*, which was more expressed in depleted *hsfA* cells and recently identified as important for the regulation of the CASP paradoxical effect (Valero et al., 2020). Noteworthy, human transcription factors involved in the immune response, such as STAT1, STAT3, NF-IL6, and NF-kB, can induce *HSP90* expression, either synergistically or antagonistically to HSF1 [reviewed in Prodromou (2016)].

Our present study results indicate that the thickening of the cell wall is a consequence of HS, and the mechanisms by which HsfA participates in this event are intertwined with the main signaling pathways that control cell integrity, including the CWI and HOG pathways. We found synthetically sick genetic interaction between *hsfA* and *mpkA* or *sakA* both under temperature and cell wall stresses, but not with the hypomorphic allele *pkcA*^*G*579*R*^ (Rocha et al., 2015), suggesting that these MAPKs act upstream *hsfA* in response to these stressors.

However, our luciferase assays indicate that PkcA and MpkA are not crucial for HsfA activation since the expression of HsfA and *hsp90P* activity were boosted during the HS and CASP treatment in the CWI pathway mutant backgrounds (Figures 6, 7). We argue that functional impairment of any CWI pathway component is sufficient to increase HsfA expression to likely activate downstream targets, and some of them could be shared with the CWI pathway. To this end, another observation of our study is that the HsfA signal was more intense in the *pkcA*^*G*579*R*^ background at both stress conditions. In contrast, the activity of *hsp90P* was more intense in the Δ*mpkA* only in the HS, given the signals recorded in the *pkcA*^*G*579*R*^ and Δ*mpkA* strains were comparable in the presence of CASP. These observations again point to the crucial role of PkcA in the early adaptation to HS as described previously (Rocha et al., 2020b). Supporting this idea, overexpression of Pkc1^*PkcA*^, but not the MAPKs Mpk1^*MpkA*^, Mkk1^*Mkk*1^, and Bck1^*Bck*1^ in *S. cerevisiae*, can suppress the Hsf1^*HsfA*^ defects in response to heat stress, meaning that the signaling emerging from Pkc1 and not Pkc1-regulated MAPK cascade was necessary for *HSF1* suppression (Imazu and Sakurai, 2005). Although a collection of kinases that phosphorylate and modulate the ScHsf1 activity (reviewed in Veri et al., 2018) is known, the enzymes that phosphorylate HsfA during the HS or cell wall stress are still unknown, thus requiring further experimentation (Figure 10).

Noteworthy, our RNA-seq indicates that HsfA is also important for the plasma membrane homeostasis in the face of a temperature upshift (Figures 8, 9). Since the fungal plasma membrane adapts to temperature changes [reviewed in Fabri et al. (2020)], our data suggested that HsfA has a repressive role in the biosynthesis of ergosterol and lipids in general, especially the unsaturated fatty acids, indicating that this transcription factor may regulate the chemical and consequently physical changes of the plasma membrane during HS. Not surprisingly, a molecular link between fatty acid synthesis and the HS response governed by Hsf1 in *C. albicans* was described previously (Leach and Cowen, 2014) and is an open field of investigation in *A. fumigatus*.

In summary, we characterized the essential HSF HsfA and demonstrated its contribution to cell wall maintenance. We observed a crosstalk between HsfA-Hsp90 and CWI and provided evidence that the kinases PkcA and MpkA are not essential for the HsfA activation during the cell wall and heat stresses. Additionally, we identified genes modulated by HsfA, including those related to HS response, cell wall biogenesis, lipid metabolism, and iron homeostasis. Our results reinforce the concept that cell wall adaptation contributes to the thermotolerance of *A. fumigatus* via an integrated mechanism encompassing HS response and the regulation of the CWI signaling pathways in fungi.

## Data Availability Statement

The datasets presented in this study can be found in online repositories. The names of the repository/repositories and accession number(s) can be found in the article/Supplementary Material.

## Author Contributions

IM planned and designed research. JF, MR, CF, and LR performed research. IM, AC, GG, LR, and MD contributed reagents/analytic tools. IM, JF, AC, LR, GG, CF, and MD analyzed and validated the data. GP conducted RNA-seq analyses. JF and IM wrote the original draft of the manuscript. All authors discussed the data, and edited and approved the manuscript.

## Conflict of Interest

MD is a Co-Founder and Chief Scientific Officer (CSO) of MicroRid Technologies Inc. The remaining authors declare that the research was conducted in the absence of any commercial or financial relationships that could be construed as a potential conflict of interest.

## Footnotes

1 http://www.bioinformatics.babraham.ac.uk/projects/fastqc/

2 http://mev.tm4.org/
