# Supplementary material for: The Heat Shock Transcription Factor HsfA Is Essential for Thermotolerance and Regulates Cell Wall Integrity in Aspergillus fumigatus
Source: Front Microbiol. 2021 Apr 9;12:656548. doi: 10.3389/fmicb.2021.656548 (PMC8062887; doi:10.3389/fmicb.2021.656548)
Supplement: Supplementary file 3 [file Data_Sheet_1.pdf]

Supplementary Material

Supplementary Figures and Tables

Supplementary Table 1: *A. fumigatus* strains used in this study.

Name	Genotype	Reference
ΔKU80 pyrG1[a]	Δ*akuB; pyrG- MAT1-1*	(da Silva Ferreira et al., 2006)
xylP::*hsfA*	*pyrG*::*xylP*::*hsfA;* Δ*akuB*	This study
pkcA^{G579R}	*pkcA*^{G579R}::*pyrG*; Δ*akuB*	(Rocha et al., 2015)
pkcA^{G579R} *pyrG-*	*pkcA*^{G579R}*;* Δ*akuB*	(Rocha et al., 2020)
pkcA^{G579R} *xylP*::*hsfA*	*pkcA*^{G579R}; *pyrG*::*xylP::hsfA*; Δ*akuB*	This study
Δ*mpkA*	Δ*mpkA::ptrA;* Δ*akuB,* PtR	(Valiante et al., 2009)
Δ*mpkA xylP*::*hsfA*	Δ*mpkA::ptrA; pyrG::xylP::hsfA;* Δ*akuB,* PtR	This study
Δ*rlmA*	Δ*rlmA::pyrG;* Δ*akuB*	(Rocha et al., 2016)
Δ*sakA*	Δ*sakA*::*hph*; Δ*akuB*, HygR	(Altwasser et al., 2015)
Δ*sakA xylP*::*hsfA*	Δ*sakA*::*hph; pyrG::xylP::hsfA;* Δ*akuB*, HygR	This study
hsp90P::luc	*hsp90P::luc::hph;* Δ*akuB,* HygR	This study
pkcA^{G579R} *hsp90P::luc*	*pkcA*^{G579R}::*pyrG*; *hsp90P::luc::hph;* Δ*akuB,* HygR	This study
Δ*mpkA hsp90P::luc*	Δ*mpkA::ptrA; hsp90P::luc::hph;* Δ*akuB,* PtR, HygR	This study
Δ*rlmA hsp90P::luc*	Δ*rlmA::pyrG; hsp90P::luc::hph;* Δ*akuB,* HygR	This study
hsfA::luc	*hsfA::luc::pyrG*; Δ*akuB*	This study
pkcA^{G579R} *hsfA::luc*	*pkcA*^{G579R}; *hsfA::luc::pyrG*; Δ*akuB*	This study
Δ*mpkA hsfA::luc*	Δ*mpkA::ptrA; hsfA::luc::pyrG*; Δ*akuB*, PtR	This study

[a]FGSC A1160: Fungal Genetics Stock Center (http://fgsc.net). PtR: pyrithiamine resistant. HygR: hygromycin resistant.

Supplementary Table 2: Primers used in this study for construction of mutant strains.

Primer name*	Sequence (5'-3')
pRS426 HsfA tet 5UTR FW	**gtaacgccagggttttcccagtcacgacg**CACTCGCCTCTGTCCA
HsfA 5UTR tet pyrG RV	gtgcctcctctcagacagaatCAATCATCTCGCGCTATCATA
pyrG FW	GGAATTCTGTCTGAGAGGAGGC
pyrG REV	GATATCGAATTCGCCTCAAAC
pyrG 200 REV	ATCGTCAAGGTTTTCCCTTTG
xylP pyrG FW	agcattgtttgaggcgaattcACTGATGCGAGCAACAG
xylP RV	GGTTGGTTCTTCGAGTCG
HsfA 2 xylP 5UTR FW	catcgactcgaagaaccaaccTTTAGCGCTCGCAGTG
HsfA 2 5UTR RV pRS426	**gcggataacaatttcacacaggaaacagc**TACAGGCGACAGACTTGA
HsfA 600 ups	TCGACGGGAGGATTTATACG
HsfA 1400 5F	**gtaacgccagggttttcccagtcacgacg**ATCAACAAGTTGAGGAGAGAGCA
HsfA ORF REV GFP	agttcttctcctttactcatAGCCTTCCTGCGCCGTTTC
GFP FW	ATGAGTAAAGGAGAAGAACT
HsfA 3F	aagagcattgtttgaggcgaattcgatatcACTTGGCTTCGAGTCAATAAAAA
HsfA 3R	**gcggataacaatttcacacaggaaacagc**CAAACACCGTCCCAAGACG
HsfA Lucif Rv	gttcttggcgtcctccatAGCCTTCCTGCGCC
Luc FW	ATGGAGGACGCCAAGAAC
Luc REV	tgcctcctctcagacagaatCTAGACGGCGATCTTGCC
HsfA 3UTR pyrG FW	gcattgtttgaggcgaattcTGTTACATGACATCTACTAACTTG
HsfA 3UTR pRS426 RV	**gcggataacaatttcacacaggaaacagc**GCCATCTACCAGCCAGT
HsfA 900 FW	TCCCCAGAGCGACATAATGT
pRS426 5UTR pyrG FW	**gtaacgccagggttttcccagtcacgacg**TCCCCTATCCGCACAG
5UTR pyrG RV	CGTGGGAATGGAGGGT
Hsp90P pyrG FW	accctccattcccacgCGATCCCTCGTTTTAGATGAT
Hsp90P RV luc	cggtgaccatggtgtttaaGATGGCGGAGGGAGA
Luc 2 FW	TTAAACACCATGGTCACCG
pyrG 3UTR pRS426 RV	**gcggataacaatttcacacaggaaacagc**CCTCCTTCTCCGCTCT
pyrG 500 ups	TGAGATACTGAGTCAGAAGA
pkcA GC FW	TTGTCATGCTCAATGTACTCACCTTGTCCCTGACTTTTGTCGCATGTCCA
Afu5g11970 3R	TCGTCATTTGTATTACCTGCCA
RlmA 600 ups	GAATGAGAAGAAAGGAGGAATGA
MpkA 600 ups	GAGCCCTGACTTCACTGCA
MpkA 5F[§]	CTCATTCCTTGTTCTGATGCG
MpkA 3R	**gcggataacaatttcacacaggaaacagc**TCAAGACTTTCCCAGTTCGG
MpkA 3' REV	GACTGTCGCAGAAATCCGCTT
MpkA FW	GCGGCAGCCATATGCTCGAGATGTCTGATCTACAGGGTC
MpkA REV	TCGGGCTTTGTTAGCAGCCGCTATTGGACATCCATCCCCCG
SakA yes FW[#]	GTCTGTCTAAGGCAATATCG

SakA yes REV[#]	CTATGGAGTGATCCCCGTCG
IM-563	GCGGCAGCCATATGCTCGAGATGATTTACTTTGTCACAGAGC
IM-566	TCGGGCTTTGTTAGCAGCCGCTATAGAAGATCGACCGC

Small bold letters indicate homology to the pRS426 flanking sequence.

Small, underlined letters indicate homology to a fragment in the cassette.

* For primers location refer to Figure S1.

§ Sequence from Valiante et al. (2009).

# Sequence from Altwasser et al. (2015).

Supplementary Table 3: Real-time PCR primers used in this study

Gene	Systematic name	Primer name	Sequence (5'-3')
hsfA	Afu5g01900	IM-q149	CGTGGTCATCCGGATCCGGATCTACTG
		IM-q150	TGCCCCGCGGTGTTC
hsp90	Afu5g04170	IM-q19	CGCCAACATGGAGCGTATC
		IM-q20	TGTAAGAGCTCATGGAGGTGTCA
hsp30	Afu6g06470	IM-q1	CGCATCGCCGGCTAA
		IM-q2	TGTCTGGGTCGGTGAATTTGT
ags2	Afu2g11270	IM-q61	TCAGGGATTGGGCTGTATGT
		IM-q62	TAGCACTTGAGAAGCCAGCA
chsG	Afu3g14420	IM-q17	AGGATGAGGGCAAAGAGGTT
		IM-q18	AAGGCGTTGCTAAAGATCCA
ppoC	Afu3g12120	IM-q77	GCTCTCGAGGCCAATGTCAA
		IM-q78	GCGAAAAATGCCAGAGAGTAGAC
sidA	Afu2g07680	IM-q37	GCAGCTCGGTGGTTGACTTC
		IM-q38	CTCGCCCGTCTCAACGTT
tubA	Afu1g10910	IM-q155	TTCCCAACAACATCCAGACC
		IM-q156	CGACGGAACATAGCAGTGAA

1.1 Supplementary Figures

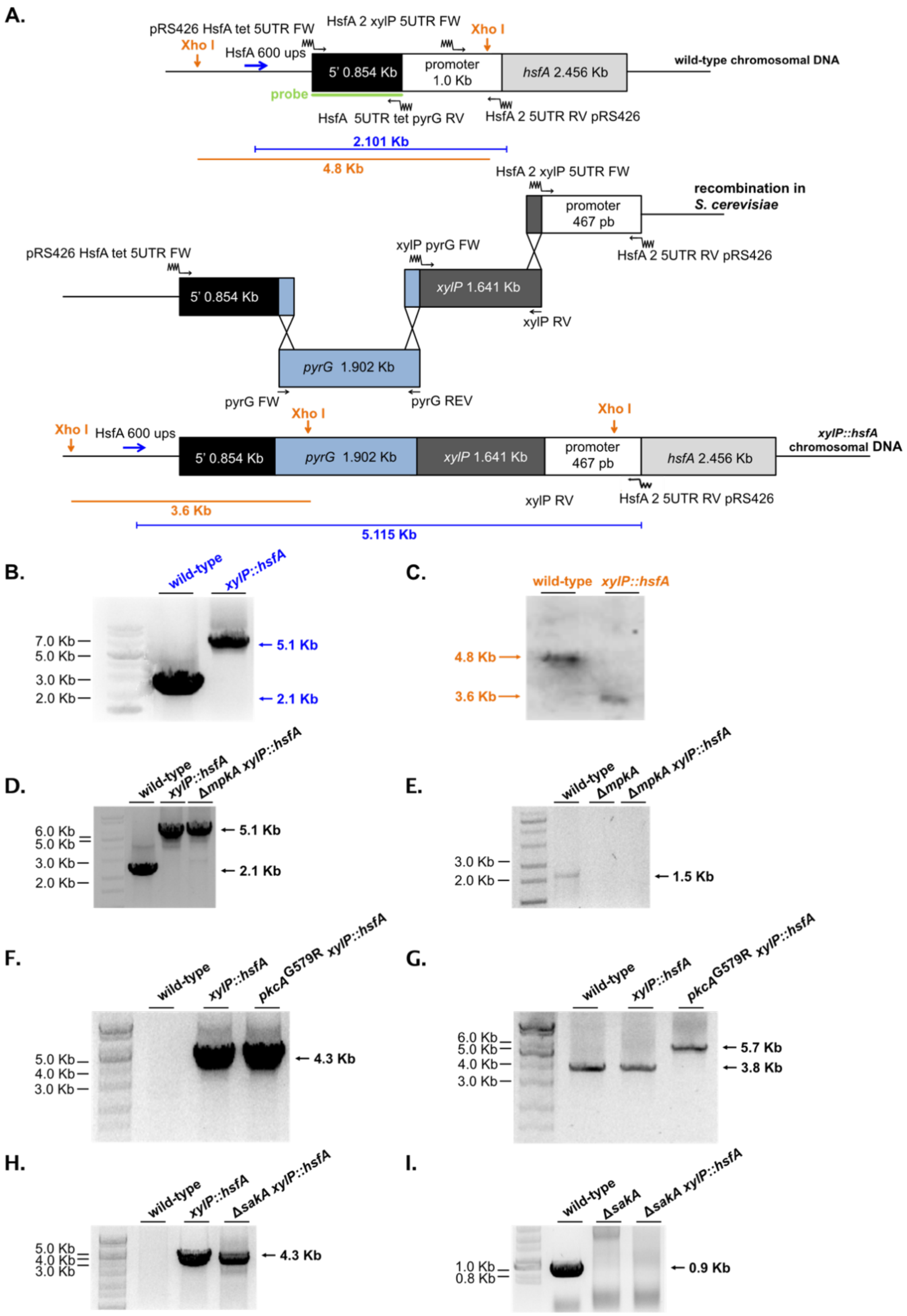

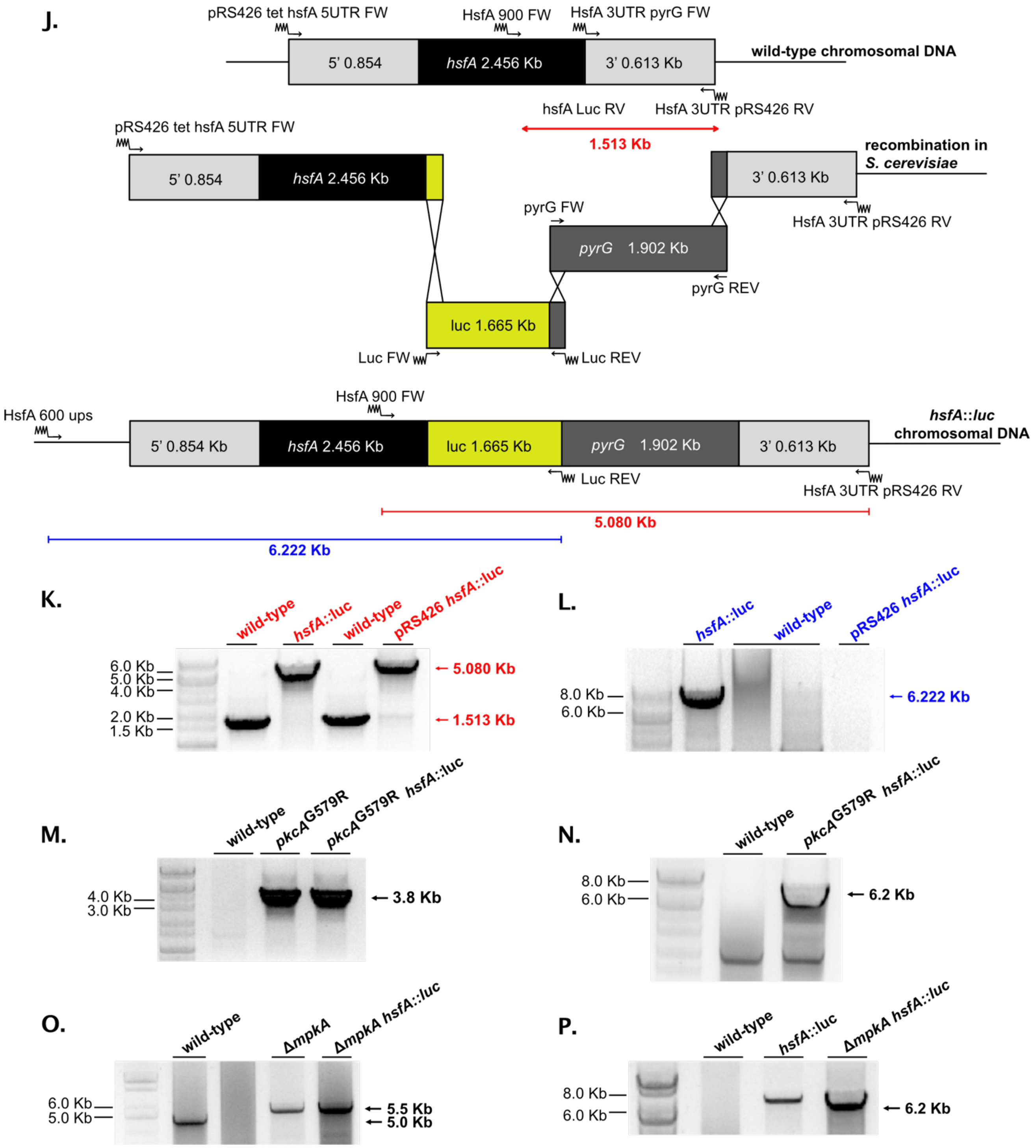
J.
pRS426 tet hsfA 5UTR FW
HsfA 900 FW
HsfA 3UTR pyrG FW
5' 0.854
hsfA 2.456 Kb
3' 0.613 Kb
wild-type chromosomal DNA
hsfA Luc RV
HsfA 3UTR pRS426 RV
1.513 Kb
pRS426 tet hsfA 5UTR FW
5' 0.854
hsfA 2.456 Kb
recombination in S. cerevisiae
3' 0.613 Kb
HsfA 3UTR pRS426 RV
pyrG FW
pyrG 1.902 Kb
pyrG REV
luc 1.665 Kb
Luc FW
Luc REV
HsfA 600 ups
HsfA 900 FW
5' 0.854 Kb
hsfA 2.456 Kb
luc 1.665 Kb
pyrG 1.902 Kb
3' 0.613 Kb
hsfA::luc chromosomal DNA
Luc REV
HsfA 3UTR pRS426 RV
5.080 Kb
6.222 Kb
K.
wild-type
hsfA::luc
wild-type
pRS426 hsfA::luc
6.0 Kb
5.0 Kb
4.0 Kb
2.0 Kb
1.5 Kb
5.080 Kb
1.513 Kb
L.
hsfA::luc
wild-type
pRS426 hsfA::luc
8.0 Kb
6.0 Kb
6.222 Kb
M.
wild-type
pkcAG579R
pkcAG579R hsfA::luc
4.0 Kb
3.0 Kb
3.8 Kb
N.
wild-type
pkcAG579R hsfA::luc
8.0 Kb
6.0 Kb
6.2 Kb
O.
wild-type
ΔmpkA
ΔmpkA hsfA::luc
6.0 Kb
5.0 Kb
5.5 Kb
5.0 Kb
P.
wild-type
hsfA::luc
ΔmpkA hsfA::luc
8.0 Kb
6.0 Kb
6.2 Kb

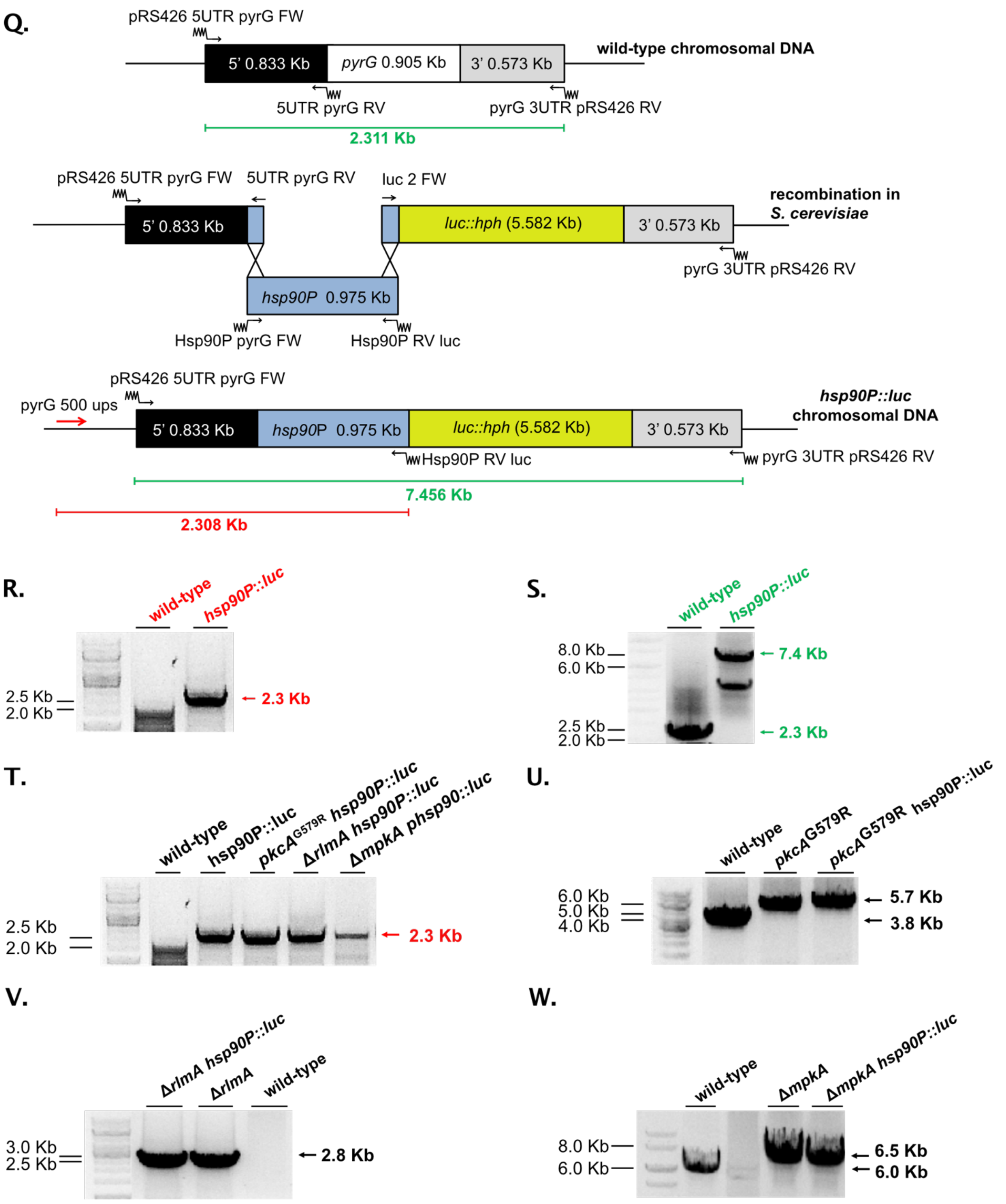

Supplementary Figure 1. Generation of mutant strains. (A) Gene replacement strategy for the *xylP::hsfA* conditional mutant construction. The promoter of xylose reductase gene from *Penicillium chrysogenum* was used to control *hsfA* transcription. The *pyrG* gene was used as a selection marker. The primer names and binding sites are indicated by arrows (primer sequences are described in Table

S2) . (B) Diagnostic PCR to evaluate the *hsfA* locus after transformation using a primer (HsfA 600 ups) located outside the cassette, as shown by blue lines and blue letters. (C) Southern blot analysis for the conditional mutant. *Xho*I-digested genomic DNA with a probe that binds specifically to the *hsfA* 5'- region indicated the predicted 3.6 Kb band in the *xylP::hsfA* mutant, as shown by orange lines. (D-I) PCRs for the validation of the *xylP::hsfA* double mutants, using the primers HsfA 600 ups and HsfA 2 5UTR RV pRS426 (D), MpkA FW and MpkA REV (E), pRS426 HsfA tet 5UTR FW and xylP REV (F,H), pkcA GC FW and Afu11970 3R (G) and IM-563 and IM-566 (I). (J) Gene replacement strategy for *hsfA::luc* strain construction. The *hsfA* genomic sequence with no stop codon was cloned in-frame with the *luc* gene in a C-terminal fusion. The *pyrG* gene was also used as a prototrophy marker. (K-L) Diagnostic PCRs to evaluate the *hsfA* locus after gene replacement, using the primers that amplify the regions shown in red (K) and in blue (L). (M-P) PCRs for the validation of the *hsfA::luc* double mutants, using the primers pkcA GC FW and pyrG REV (M), HsfA 600 ups and Luc REV (N,P) and MpkA 5F and MpkA 3R (O). (Q) Gene replacement strategy for *hsp90P::luc* strain construction. The promoter region of *hsp90* was cloned in-frame with the *luc* gene within the *pyrG* locus. The hygromycin resistance gene was used as a selection marker. (R-S) Diagnostic PCRs to evaluate the *pyrG* locus after gene replacement, using the primers that amplify the regions shown in red (R) and in green (S). (T-W) PCRs for the validation of the *hsp90P::luc* double mutants, using the primers pyrG 500 ups and Hsp90P RV luc (T), pkcA GC FW and Afu5g11970 3R (U), RlmA 600 ups and pyrG 200 REV (V) and MpkA 600 ups and MpkA 3R (W).

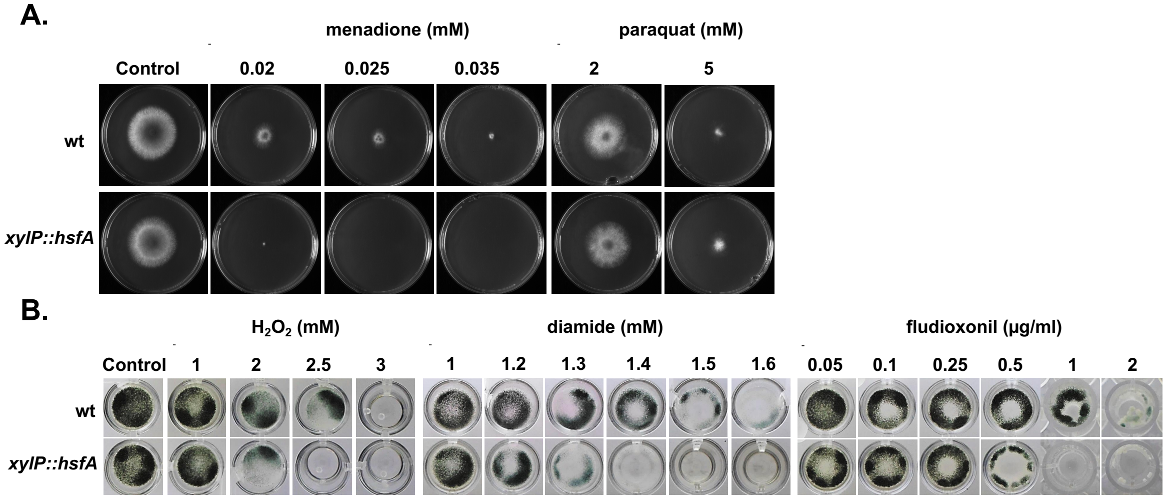

Supplementary Figure 2. *xylP::hsfA* mutant is sensitive to oxidative-damaging agents and to the fungicide fludioxonil. 1×10^4 conidia of wild-type and mutant strains were inoculated in 20 ml of MM (Petri dishes) supplemented with xylose 0.06% and varying concentration of menadione or paraquat (A), or 200 µl of MM (96 well plates) supplemented with xylose 0.06% and varying concentration of H_2O_2, fludioxonil or diamide (B). Plates were incubated at 37ºC for 72 h and photographed.

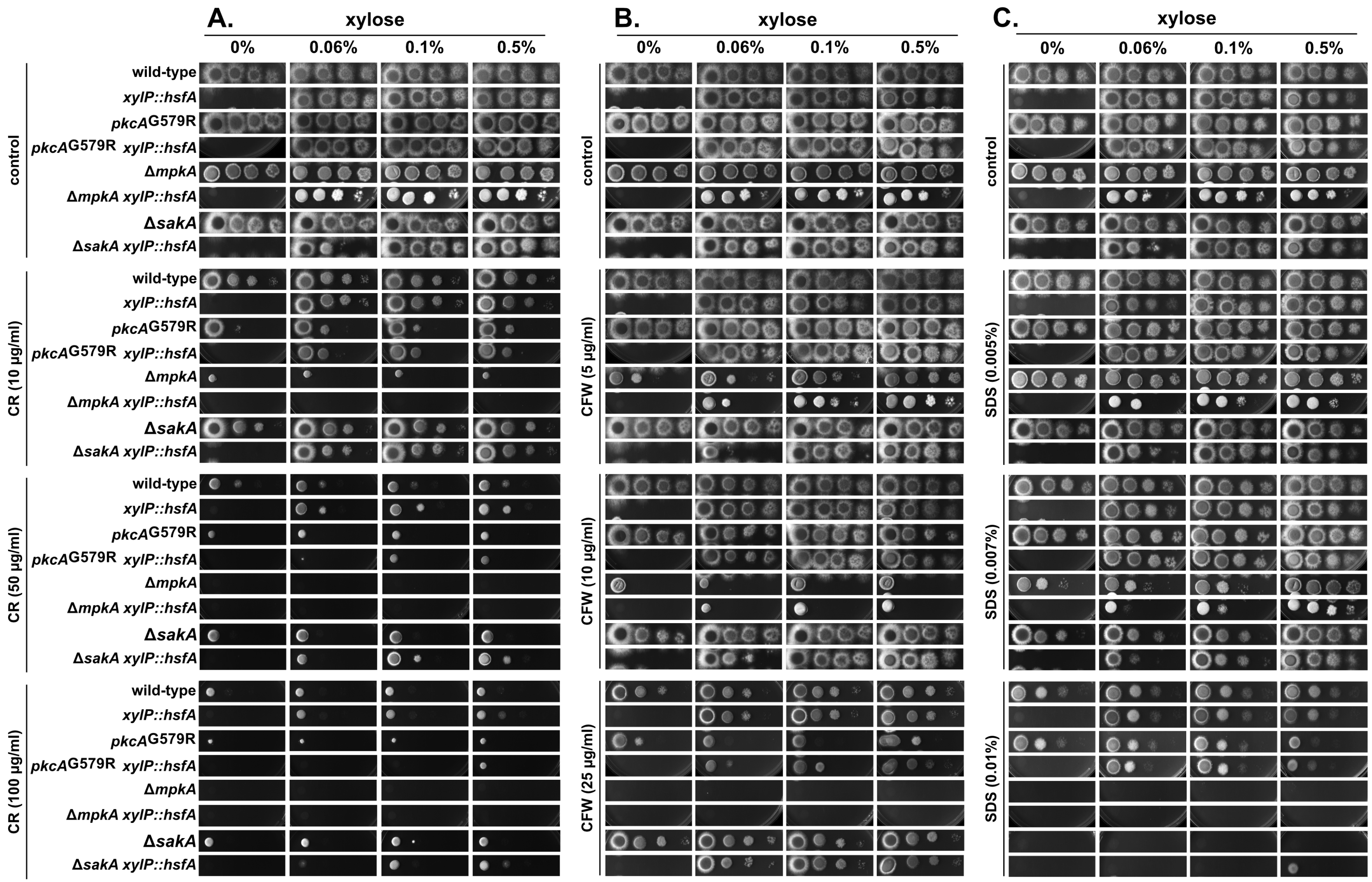

Supplementary Figure 3. *hsfA* genetically interacts with *mpkA* and *sakA* in the presence of CR, CFW and SDS. Conidia (10-fold dilutions) of the relevant strains were inoculated onto solid MM supplemented with different concentrations of xylose and increasing concentrations of CR (A), CFW (B) and SDS (C). The plates were incubated at 37°C for 48 h.

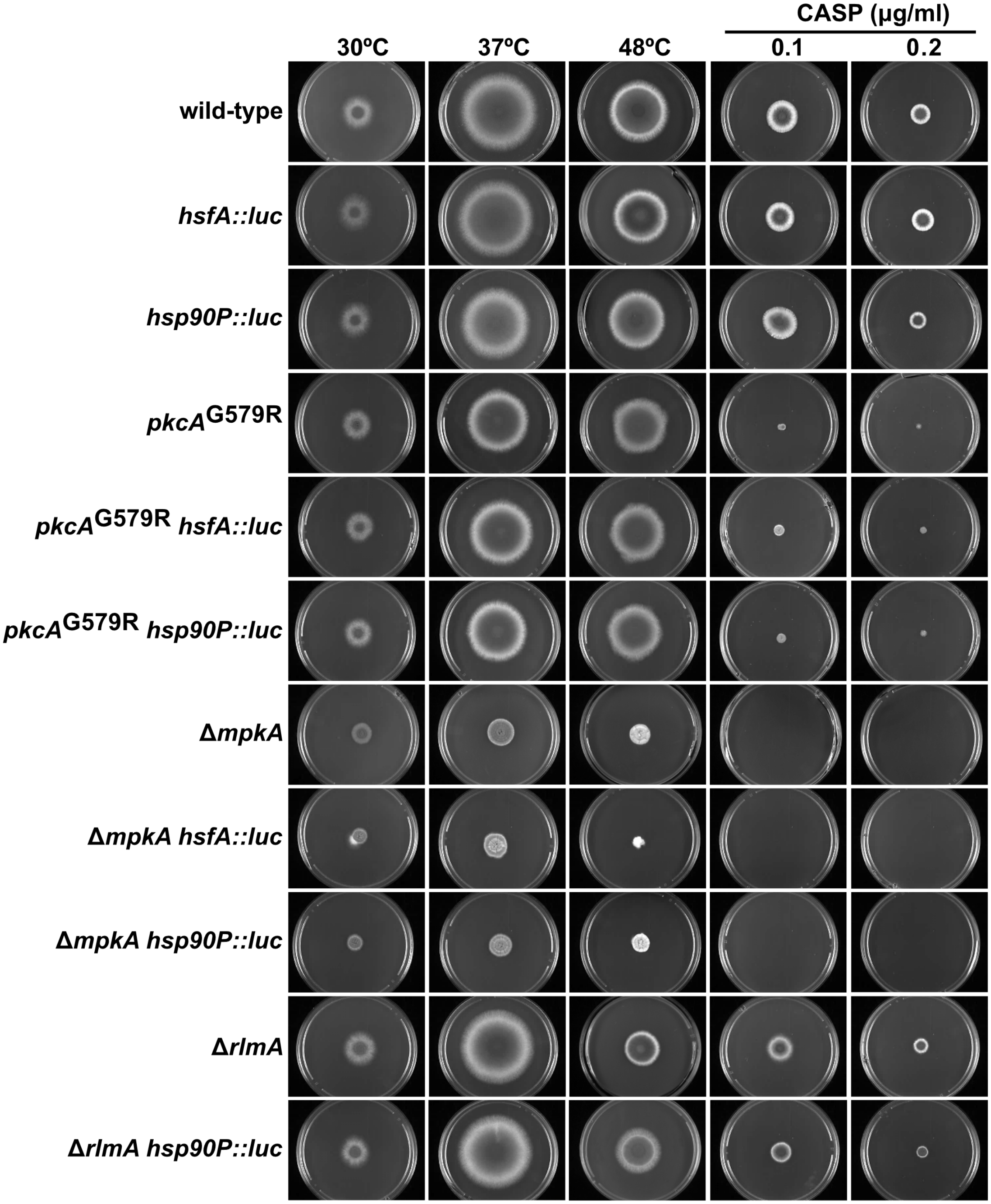

Supplementary Figure 4. Growth phenotype of the *hsp90P::luc* and *hsfA::luc* single and double mutants. 1×10^4 conidia of each strain were inoculated on solid MM and incubated at the indicated temperatures for 96 h. Plates with different concentrations of CASP were incubated at 37ºC for 96 h.

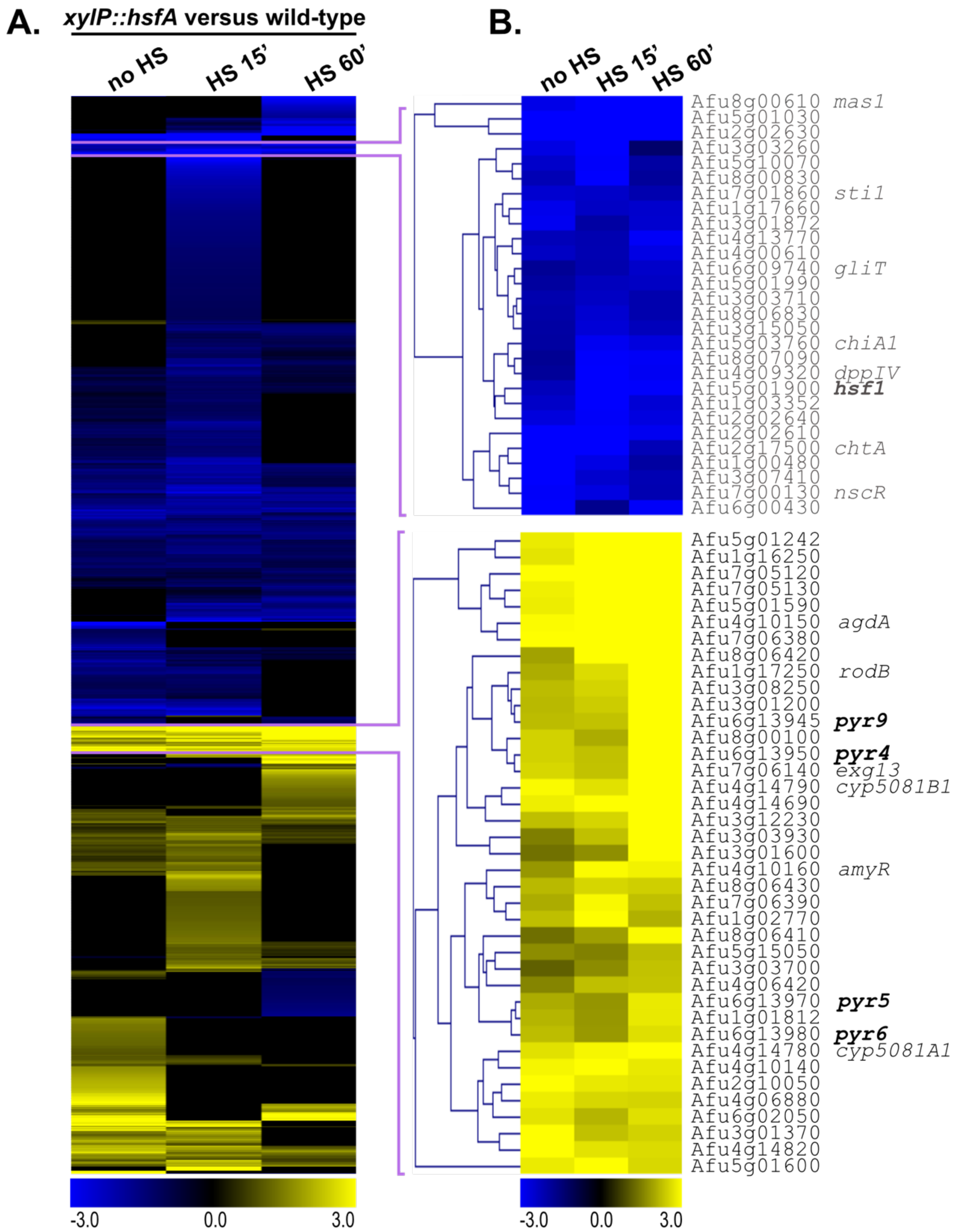

Supplementary Figure 5. The *A. fumigatus* transcriptome is modified when *hsfA* is repressed. (A) Hierarchical clustering analysis showing the differentially expressed genes in the comparison *xylP::hsfA versus* wild-type at no HS condition (30ºC) or after 15 min and 60 min of HS. Only genes with modulation of $\log_2 FC \geq 1.0$ or $\log_2 FC \leq -1.0$ in at least one time point were selected. (B) Sections showing the most constitutively upregulated and downregulated genes are highlighted. The heat maps were created in the Multiple Experiment Viewer (MeV) platform, using hierarchical clustering and Euclidean Distance with average linkage clustering.

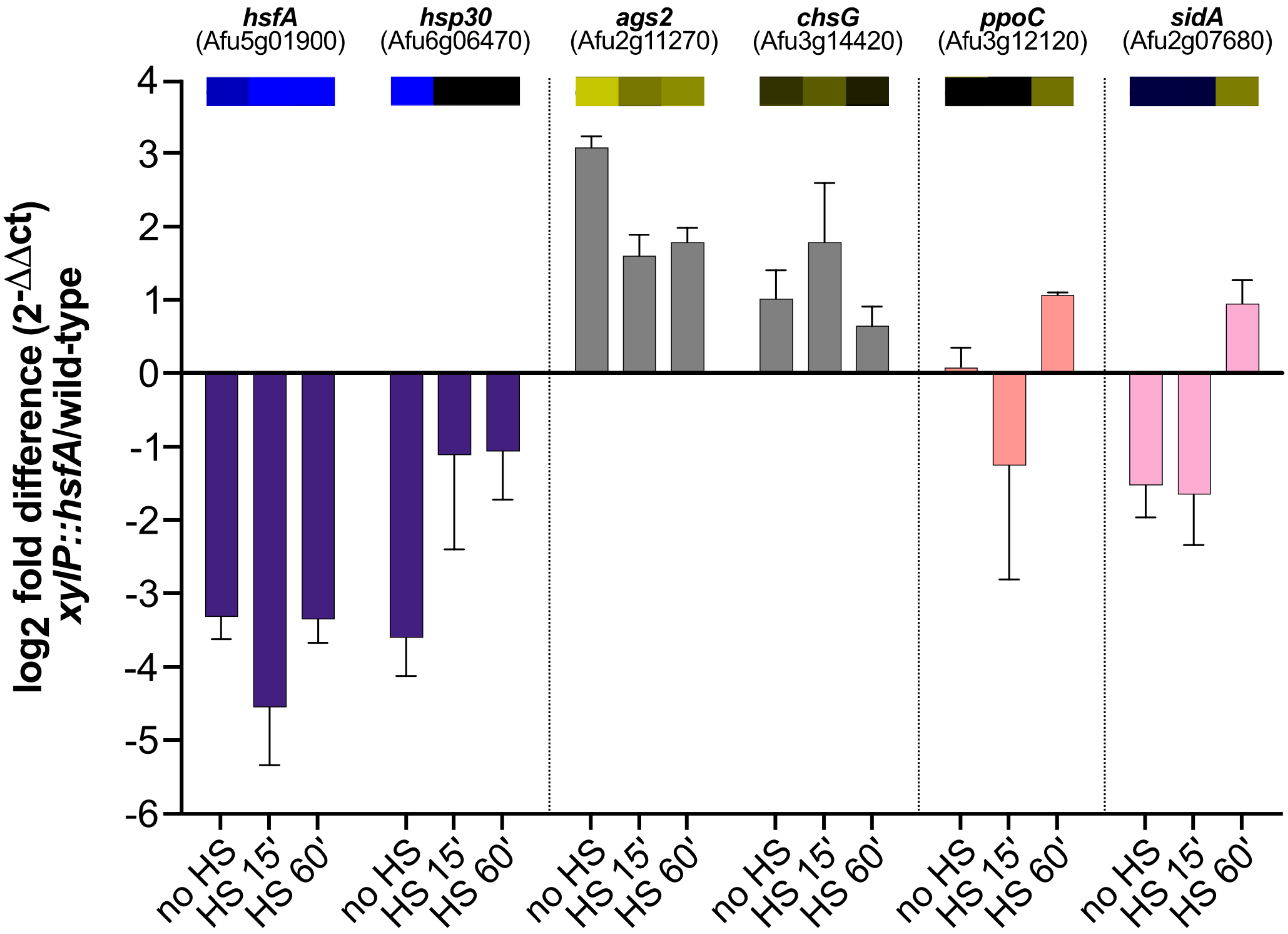

Supplementary Figure 6. RT-qPCR of selected genes differentially expressed in the RNAseq analysis. The different colors in the bars separated by dashed lines indicate a different GO category as shown in Figure 9, *i. e.*, heat shock response, chaperone activity and protein folding; cell wall biosynthesis and organization; lipid metabolism; and iron metabolism, respectively. The strains were grown in liquid MM supplemented with xylose 1% for 24 h at 30ºC. Subsequently, mycelia were washed twice with MM and incubated for 4 h at 30ºC in MM for *hsfA* repression. HS was induced by transferring the mycelia to fresh pre-heated MM for 15 and 60 min at 48ºC. The abundance of each gene was divided by the abundance of the normalizer *tubA*. The results are expressed as log2 of the ratio *xylP::hsfA*/wild-type for each time point. Mean ± SD (n = 3) is shown. For comparison, the heat maps at the top show the RNAseq results for each gene.

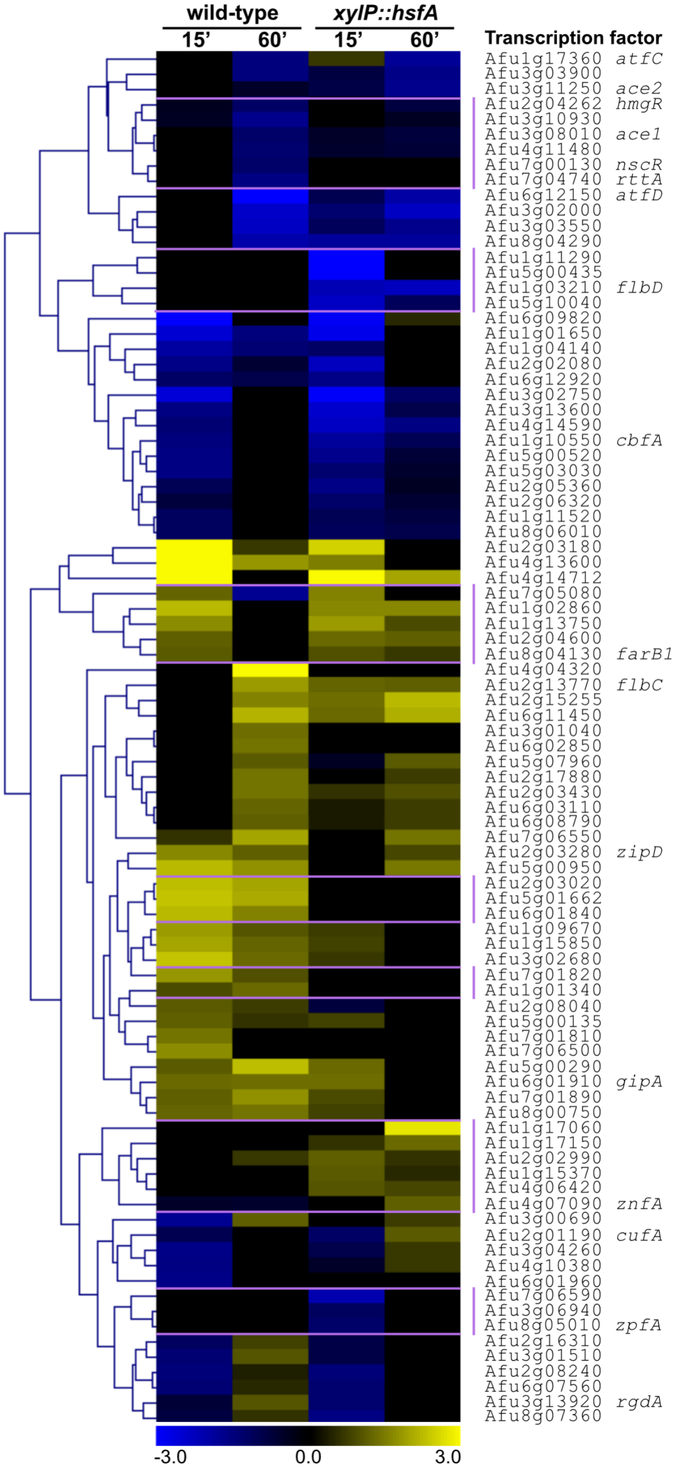

Supplementary Figure 7. Transcription factors that display differential expression upon HS and are dependent on HsfA. The data set from Table S4 was manually inspected, searching for genes encoding transcription factors. Only genes with modulation of $\log_2FC \geq 1.0$ or $\log_2FC \leq -1.0$ ($p \leq 0.01$) in at least one time-point (15 min or 60 min of HS) for either strain were selected. A heat map depicting the $\log_2FC$ of differentially expressed genes was created in the Multiple Experiment Viewer (MeV) platform, using hierarchical clustering and Euclidean Distance with average linkage clustering.

References

Altwasser, R., Baldin, C., Weber, J., Guthke, R., Kniemeyer, O., Brakhage, A.A., et al. (2015). Network Modeling Reveals Cross Talk of MAP Kinases during Adaptation to Caspofungin Stress in *Aspergillus fumigatus*. *PLoS One* 10(9)**,** e0136932. doi: 10.1371/journal.pone.0136932.

da Silva Ferreira, M.E., Kress, M.R., Savoldi, M., Goldman, M.H., Hartl, A., Heinekamp, T., et al. (2006). The *akuB*^{KU80} mutant deficient for nonhomologous end joining is a powerful tool for analyzing pathogenicity in *Aspergillus fumigatus*. *Eukaryot Cell* 5(1)**,** 207-211. doi: 10.1128/EC.5.1.207-211.2006.

Rocha, M.C., Fabri, J.H., Franco de Godoy, K., Alves de Castro, P., Hori, J.I., Ferreira da Cunha, A., et al. (2016). Aspergillus fumigatus MADS-Box Transcription Factor *rlmA* Is Required for Regulation of the Cell Wall Integrity and Virulence. *G3 (Bethesda)* 6(9)**,** 2983-3002. doi: 10.1534/g3.116.031112.

Rocha, M.C., Godoy, K.F., de Castro, P.A., Hori, J.I., Bom, V.L., Brown, N.A., et al. (2015). The *Aspergillus fumigatus pkcA*^{G579R} Mutant Is Defective in the Activation of the Cell Wall Integrity Pathway but Is Dispensable for Virulence in a Neutropenic Mouse Infection Model. *PLoS One* 10(8)**,** e0135195. doi: 10.1371/journal.pone.0135195.

Rocha, M.C., Minari, K., Fabri, J., Kerkaert, J.D., Gava, L.M., da Cunha, A.F., et al. (2020). *Aspergillus fumigatus* Hsp90 interacts with the main components of the cell wall integrity pathway and cooperates in heat shock and cell wall stress adaptation. *Cell Microbiol***,** e13273. doi: 10.1111/cmi.13273.

Valiante, V., Jain, R., Heinekamp, T., and Brakhage, A.A. (2009). The MpkA MAP kinase module regulates cell wall integrity signaling and pyomelanin formation in *Aspergillus fumigatus*. *Fungal Genet Biol* 46(12)**,** 909-918. doi: 10.1016/j.fgb.2009.08.005.
